# Optimizations for identifying reference genes in bone and cartilage bioengineering

**DOI:** 10.1186/s12896-021-00685-8

**Published:** 2021-03-17

**Authors:** Fei Xiong, Xiangyun Cheng, Chao Zhang, Roland Manfred Klar, Tao He

**Affiliations:** 1 Department of Sports Medicine, Wuxi 9th People’s Hospital affiliated to Soochow University, Wuxi, Jiangsu China; 2 Department of Orthopedics, Renji Hospital, School of Medicine, Shanghai Jiaotong University, Shanghai, China; 3 Department of Orthopedics, Physical Medicine and Rehabilitation, University Hospital of Munich (LMU), Munich, Germany

**Keywords:** RT-qPCR, Reference gene set, Osteogenesis, Chondrogenesis, Bioengineering

## Abstract

**Background:**

Reverse transcription quantitative real-time polymerase chain reaction (RT-qPCR) remains one of the best-established techniques to assess gene expression patterns. However, appropriate reference gene(s) selection remains a critical and challenging subject in which inappropriate reference gene selction can distort results leading to false interpretations. To date, mixed opinions still exist in how to choose the most optimal reference gene sets in accodrance to the Minimum Information for Publication of Quantitative Real-Time PCR Experiments (MIQE) guideline. Therefore, the purpose of this study was to investigate which schemes were the most feasible for the identification of reference genes in a bone and cartilage bioengineering experimental setting. In this study, rat bone mesenchymal stem cells (rBMSCs), skeletal muscle tissue and adipose tissue were utilized, undergoing either chondrogenic or osteogenic induction, to investigate the optimal reference gene set identification scheme that would subsequently ensure stable and accurate interpretation of gene expression in bone and cartilage bioengineering.

**Results:**

The stability and pairwise variance of eight candidate reference genes were analyzed using geNorm. The V_0.15_- vs. V_min_-based normalization scheme in rBMSCs had no significant effect on the eventual normalization of target genes. In terms of the muscle tissue, the results of the correlation of NF values between the V_0.15_ and V_min_ schemes and the variance of target genes expression levels generated by these two schemes showed that different schemes do indeed have a significant effect on the eventual normalization of target genes. Three selection schemes were adopted in terms of the adipose tissue, including the three optimal reference genes (Opt_3_), V_0.20_ and V_min_ schemes, and the analysis of NF values with eventual normalization of target genes showed that the different selection schemes also have a significant effect on the eventual normalization of target genes.

**Conclusions:**

Based on these results, the proposed cut-off value of Vn/n + 1 under 0.15, according to the geNorm algorithm, should be considered with caution. For cell only experiments, at least rBMSCs, a Vn/n + 1 under 0.15 is sufficient in RT-qPCR studies. However, when using certain tissue types such as skeletal muscle and adipose tissue the minimum Vn/n + 1 should be used instead as this provides a far superior mode of generating accurate gene expression results. We thus recommended that when the stability and variation of a candidate reference genes in a specific study is unclear the minimum Vn/n + 1 should always be used as this ensures the best and most accurate gene expression value is achieved during RT-qPCR assays.

**Supplementary Information:**

The online version contains supplementary material available at 10.1186/s12896-021-00685-8.

## Background

The successful re-formation of bone and cartilage remains an unsolved riddle to achieve clinically. Whilst many new bioengineering concepts have shown great potential to possibly someday replace the golden standard utilizing autografts, bone or cartilage [1–3], their translatability clinically remain problematic. To interpret and manipulate the nature’s biological defined processes successful for clinical applications, detailed and accurate assays in genes expression patterns and modulatory mechanism are a critical fundamental knowledge that if not properly defined will continue to generate more questions than answers. Reverse transcription quantitative real-time polymerase chain reaction (RT-qPCR) has emerged as one of the fundamental techniques to properly solve the questions sought for bioengineering principles [4]. If utilized properly, it has shown to generate reliable, comparable and reproducible gene expression information on how tissues respond during bioengineering [5] making RT-qPCR a benchmark for gene analysis [4, 6] but also a critical validation tool to support NextGen sequencing and microarray assay results [7, 8]. Nevertheless, improper optimization and standardization have shown to significantly affect the variability of gene expression results generated by RT-qPCR thereby impairing the reproducibility that subsequently compromises the translation efficiency of present bioengineering techniques [4, 9–12].

To prevent the misinterpretation of results caused by non-standard experimental procedures and details [13], the Minimum Information for Publication of Quantitative Real-Time PCR Experiments (MIQE) guidelines was established, which targets the promotion of reliability of results including the consistency and transparency between laboratories [9, 14–16]. Particularly, the selection of reference genes has drawn considerable attention, because the expressions of these so-called “ideal” non-variant genes can become unstable under certain conditions [15, 17, 18]. Hence, the proper selection and combination of multiple reference genes was established to minimize the instability and variation [9]. Additionally, various methodology articles have made further efforts to ensure the stability and optimal quantity of reference genes to obtain accurately reproducible data, providing considerable impetus towards perfecting RT-qPCR [19–22]. Subsequent bone and cartilage bioengineering studies have suggested that the stability and normalization quantity of reference genes should not only be determined by cell or tissue type but should also be re-optimized for experiments under different processing conditions [23, 24], which has provided an extra step in perfecting the understanding of the MIQE guidelines.

GeNorm, a bioinformatics tool, is commonly used to find the most stable reference genes and determine the proper quantity by calculating the M value, normalization factor (NF) and the pairwise variation (V_n/n + 1_-score) [15]. The value of V_n/n + 1_ with 0.15 (V_0.15_) was generally accepted as the cut-off for choosing optimal number of reference genes, below which the participation of more reference genes was thought redundant [15]. Besides the V_n/n + 1_-score-based scheme, the geNorm algorithm also provides an alternative to select the best three reference genes based on geNorm results. Moreover, other schemes were raised due to the limitation of the V_0.15_ method [25–27]. In the study performed by Ayers et al. [25] and Hosseini et al. [26], 0.20 was set as a trade-off where in some tissue types (e.g. adipose tissue) the minimum V_n_/n + 1-score (Vmin) was higher than 0.15 [18, 19]. To select stable reference genes for a cell-based study, Lee et al. [28] assessed the stability of twelve candidate genes across experimental conditions by geNorm analysis, finally abandoned the strategy of selecting the three most stably expressed reference genes and instead adopted the V_min_ strategy. Hence, debates persist on the schemes for choosing reference genes to improve the accuracy of RT-qPCR assays [25, 27–30].

Therefore, this study sought to investigate which schemes (V_0.15_-, V_0.20_-, V_Opt3_- and V_min_-based normalization schemes) were the most feasible for the identification of reference genes in bone and cartilage bioengineering experiments, as this is our primary research direction. Our hypothesis was that identification of reference genes based on V_min_ was the optimal scheme for the normalization of RT-qPCR, thereby gaining accurate and reliable gene expression data for bone and cartilage bioengineering.

## Results

### V_0.15_- vs. V_min_-based normalization scheme for analyzing gene expression data in rBMSCs

The correlation of NF values between the V_0.15_ and V_min_ schemes was analyzed and the variance of target genes expression levels generated by these two schemes was compared in rat bone mesenchymal stem cells (rBMSCs).

**Fig. 1:**
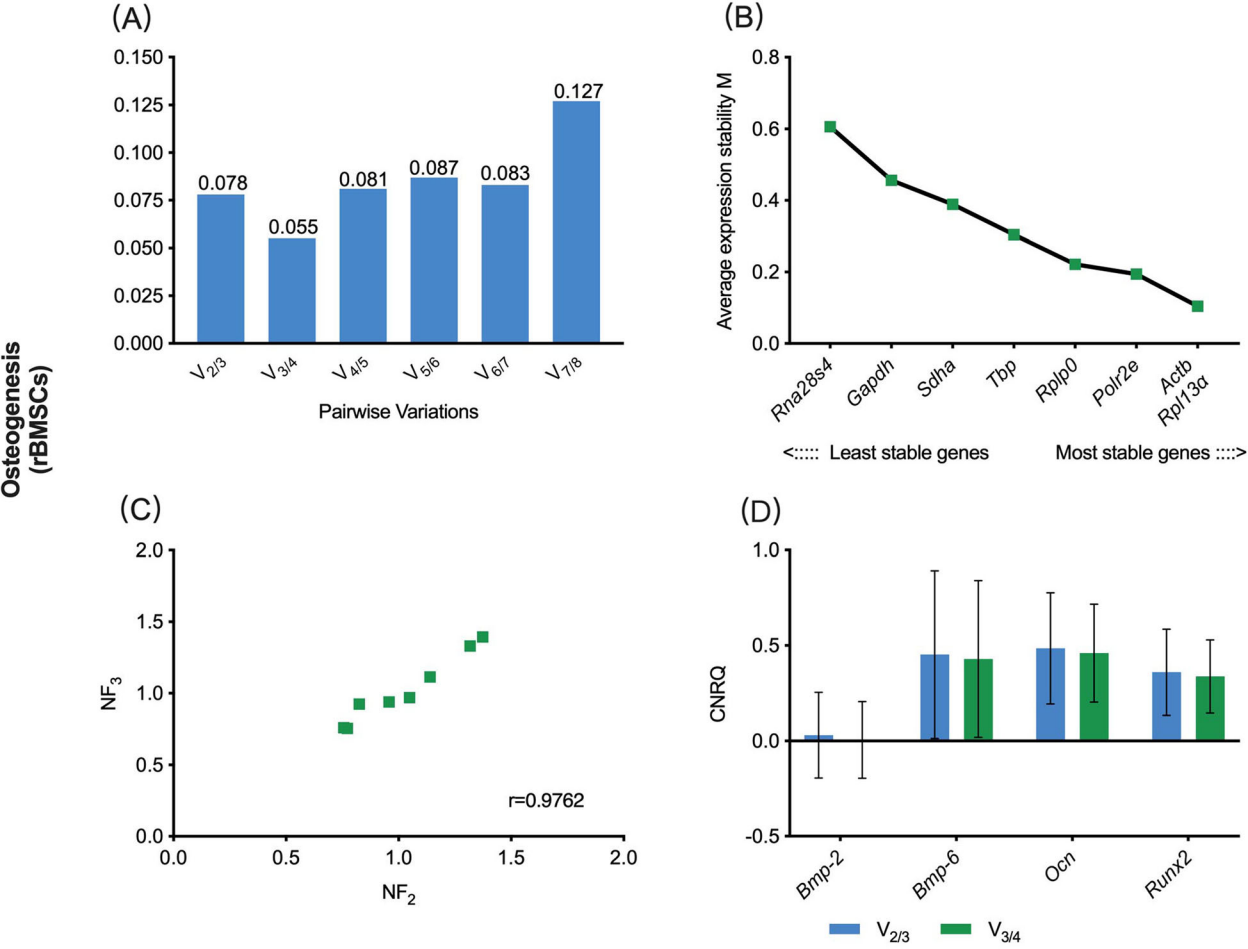
V_0.15_- vs. V_min_-based normalization scheme for gene expression assay in rBMSCs undergoing osteogenic differentiation. **a** Pairwise variation (V_n/n + 1_) analysis between NF_*n*_ and NF_*n* + 1_ to determine the optimal number of reference genes required for accurate normalization, V_0.15_ = 0.078, V_min_ = 0.055; **b** The sequencing of eight candidate reference genes based on M value; **c** Scatter plots of NFs between V_0.15_ and V_min_-based scheme (*r* = Spearman rank correlation coefficient); **d** The comparison of target gene expression levels (CNRQ) using either the V_0.15_ or V_min_-based scheme. rBMSCs, rat bone marrow mesenchymal stem cells; NF, normalization factor; CNRQ, calibrated normalized relative quantity

In the osteogenic sub-study, the V_0.15_ was 0.078, while the V_min_ was 0.055 **(**Fig. 1a). Hence, combining the sequencing of eight candidate reference genes based on M-value, the V_0.15_-based reference gene set contained *ribosomal protein L13α* (*Rpl13α*) and *actin beta* (*Actb*), while the V_min_-based reference gene set contained *Rpl13α*, *Actb* and *RNA polymerase II subunit e* (*Polr2e*) **(**Fig. 1b). The r- value in the Spearman rank correlation analysis between NF_V0.15_ and NF_Vmin_ was 0.9762 **(**Fig. 1c), which showed a very strong correlation between these two schemes in terms of rBMSCs. The variance of target genes expression levels using these two schemes was then compared. The osteogenic-related target genes, including *bone morphogenetic protein-2* (*Bmp-2*), *Bmp-6*, *osteocalcin* (*Ocn*) and *runt-related transcription factor 2* (*Runx2*) were normalized to the two reference gene sets, and the calibrated normalized relative quantity (CNRQ) values were obtained. The CNRQ values of all the investigated target genes, either using the V_0.15_ or V_min_ scheme, did not show a significant difference (*P* > 0.05) **(**Fig. 1d**)**, revealing that the two reference genes identification schemes had no significant effect on the eventual normalization of target genes.

**Fig. 2:**
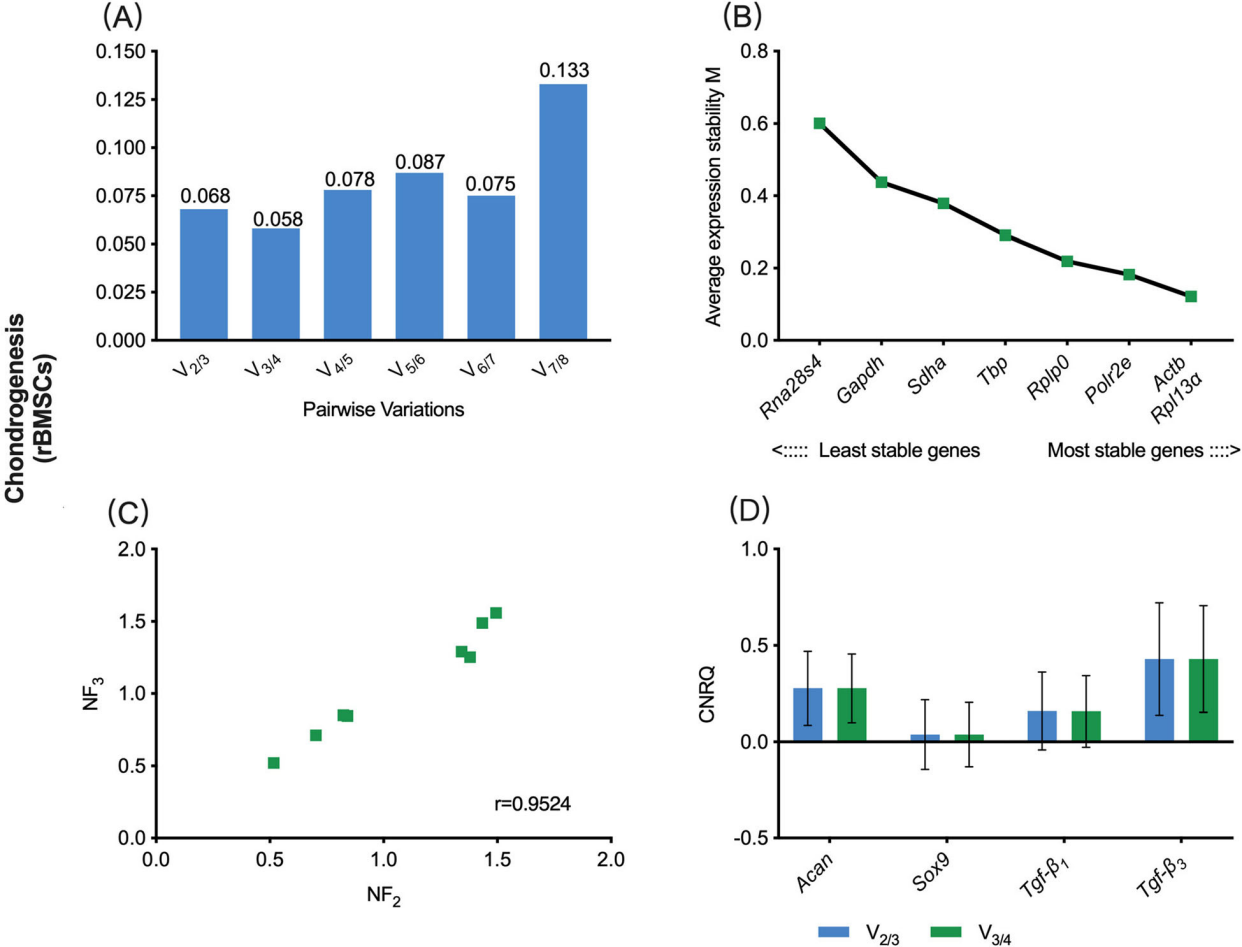
V_0.15_- vs. V_min_-based normalization scheme for gene expression assay in rBMSCs undergoing chondrogenic differentiation. **a** Pairwise variation (V_n/n + 1_) analysis between NF_n_ and NF_n + 1_ to determine the optimal number of reference genes required for accurate normalization, V_0.15_ = 0.068, V_min_ = 0.058; **b** The sequencing of eight candidate reference genes based on M value; **c** Scatter plots of NFs between V_0.15_ and V_min_-based scheme (*r* = Spearman rank correlation coefficient). **b** The comparison of target gene expression levels (CNRQ) using either the V_0.15_ or V_min_-based scheme. rBMSCs, rat bone marrow mesenchymal stem cells; NF, normalization factor; CNRQ, calibrated normalized relative quantity

In the chondrogenic sub-study, V_0.15_ was 0.068, while V_min_ was 0.058 **(**Fig. 2a**)**. Hence, combining the sequences of eight candidate reference genes based on the M-value, the V_0.15_-based reference gene set contained *Actb* and *Rpl13α*, and The V_min_-based reference gene set contained *Rpl13α*, *Actb* and *Polr2e* **(**Fig. 2b). The r-value in the Spearman rank correlation analysis between NF_V0.15_ and NF_Vmin_ was 0.9524 **(**Fig. 2c). These results showed that there was a strong correlation between these two selection schemes. The chondrogenic-related target genes, including *aggrecan (Acan), Sex determining region Y-box 9 (Sox9), transforming growth factor beta 1 (Tgf-β*_*1*_*) and Tgf-β*_*3*_, were normalized to the two reference gene sets. CNRQ values showed that the relative expression levels of *Acan*, *Sox9*, *Tgf-β*_*1*_ and *Tgf-β*_*3*_ did not show a significant deference between the V_0.15_- and V_min_- based CNRQ values (*P* > 0.05), which revealed that different selection schemes had no significant effect on the eventual normalization of target genes **(**Fig. 2d).

### V_0.15_- vs. V_min_-based normalization scheme for analyzing gene expression data in skeletal muscle tissue

The correlation of NF values between the V_0.15_ and V_min_ schemes was analyzed and the variance of target genes expression levels generated by these two schemes was compared in terms of the muscle tissue.

**Fig. 3:**
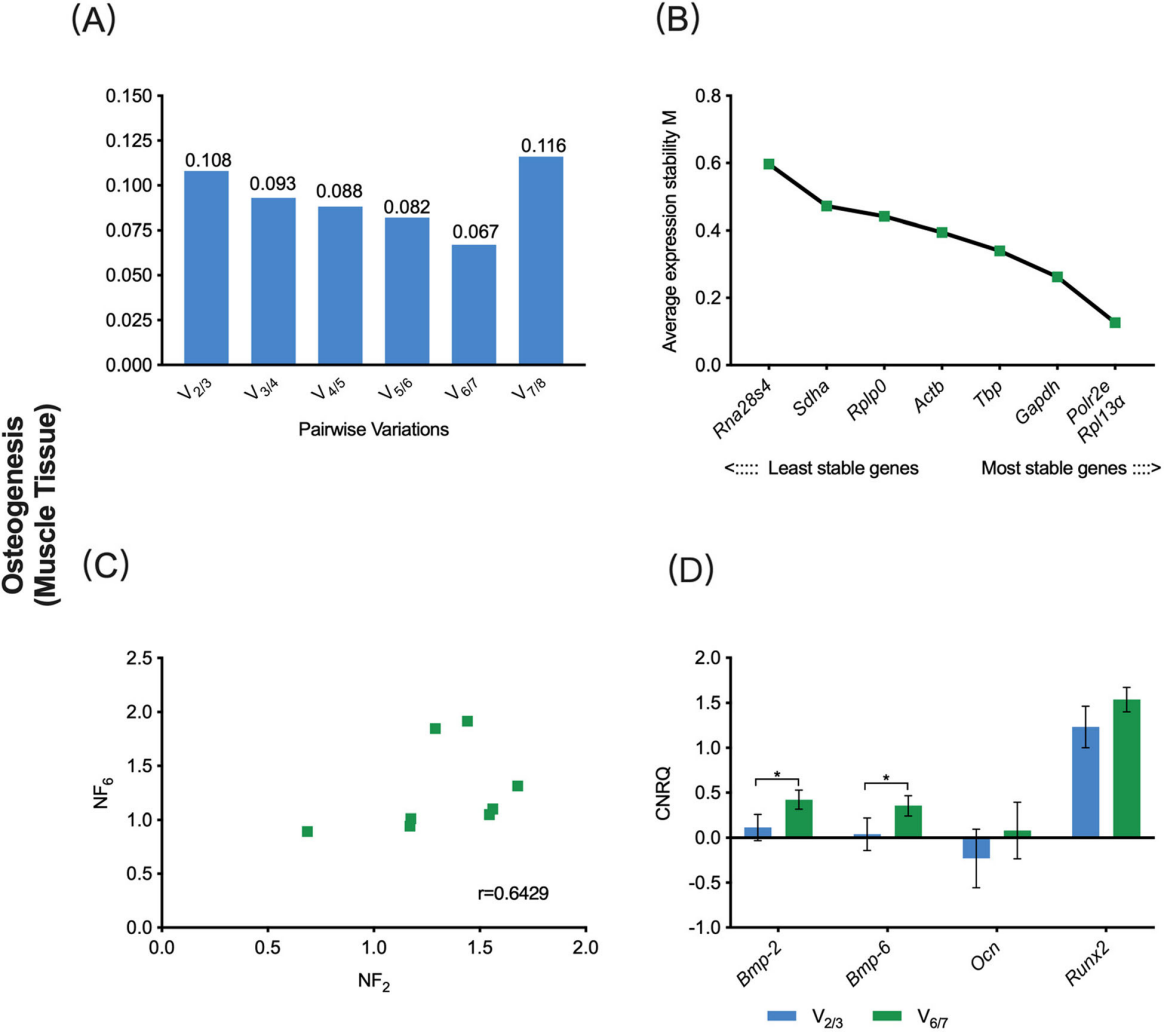
V_0.15_- vs. V_min_-based normalization scheme for gene expression assay on muscle tissue undergoing osteogenic differentiation. **a** Pairwise variation (V_n/n + 1_) analysis between NF_n_ and NF_n + 1_ to determine the optimal number of reference genes required for accurate normalization, V_0.15_ = 0.108, V_min_ = 0.067; **b** The sequencing of eight candidate reference genes based on M value; **c** Scatter plots of NFs between V_0.15_ and V_min_-based scheme (*r* = Spearman rank correlation coefficient). **d** The comparison of target gene expression levels (CNRQ) using either the V_0.15_ or V_min_-based scheme. NF, normalization factor; CNRQ, calibrated normalized relative quantity

In the osteogenic sub-study, the V_0.15_ was 0.108, while V_min_ was 0.067 **(**Fig. 3a). Hence, combining the sequencing of eight candidate reference genes based on M-value, the V_0.15_-based reference gene set contained *Polr2e* and *Rpl13α*, while the V_min_-based reference gene set contained *Rpl13α*, *Polr2e*, *glyceraldehyde 3-phosphate dehydrogenase* (*Gapdh*), *TATA-binding protein* (*Tbp*), *Actb* and *ribosomal protein lateral stalk subunit P0* (*Rplp0*) **(**Fig. 3b). The *r*-value in the Spearman rank correlation analysis between NF_V0.15_ and NF_Vmin_ was 0.6429 **(**Fig. 3c). This result showed that there was no strong correlation between these two selection schemes. The osteogenic-related target genes, including *Bmp-2*, *Bmp-6*, *Ocn* and *Runx2*, were normalized to the two reference gene sets. The relative expression levels of *Ocn* and *Runx2* showed no significant deference between the V_0.15_- and V_min_- based CNRQ values (*P* > 0.05), while the results of *Bmp-2* and *Bmp-6* showed significant deference (*P* < 0.05), which indicated that different schemes may have a significant effect on the eventual normalization of specific target genes **(**Fig. 3d).

**Fig. 4:**
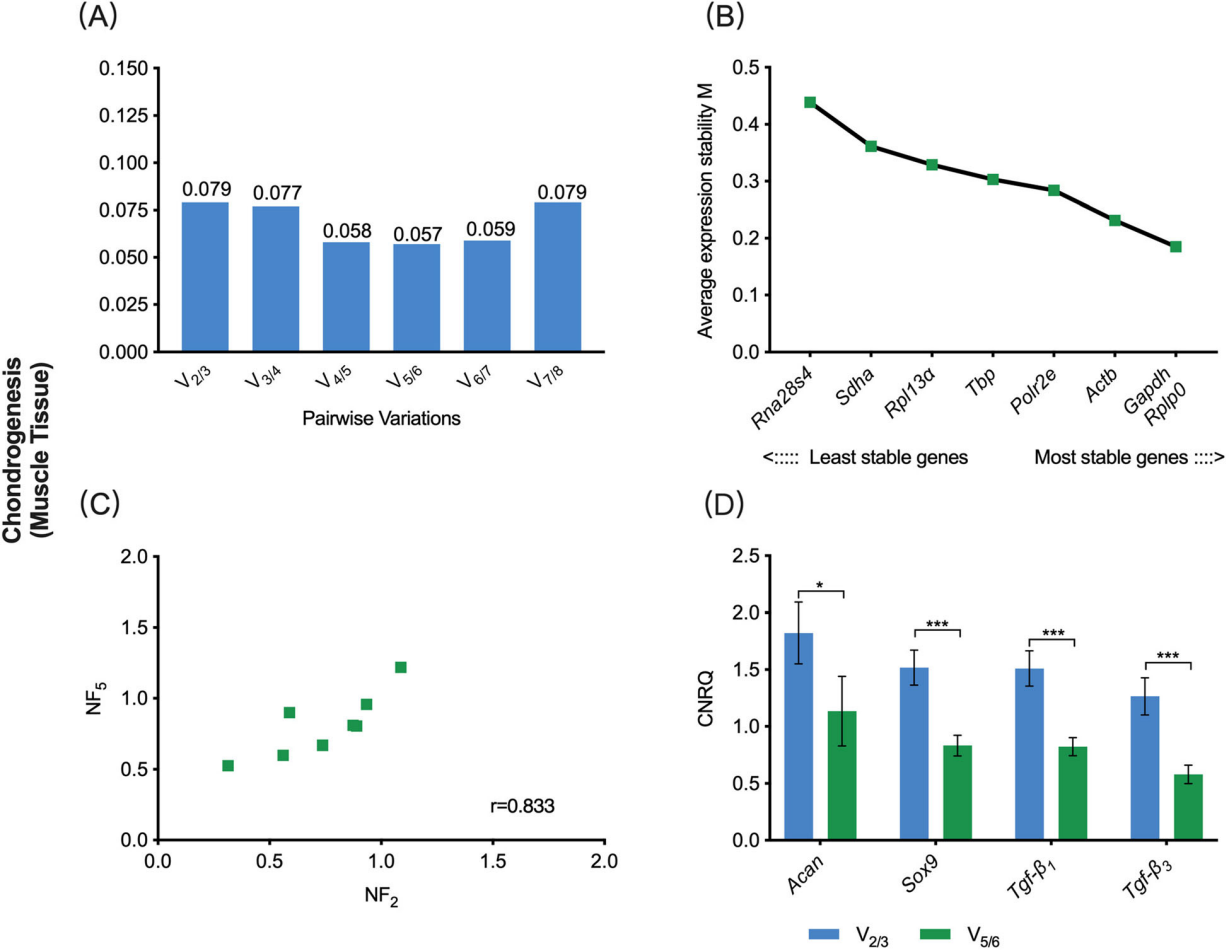
V_0.15_- vs. V_min_-based normalization scheme for gene expression assay on muscle tissue undergoing chondrogenic differentiation. **a** Pairwise variation (V_n/n + 1_) analysis between NF_n_ and NF_n + 1_ to determine the optimal number of reference genes required for accurate normalization, V_0.15_ = 0.079, V_min_ = 0.057; **b** The sequencing of eight candidate reference genes based on M value; **c** Scatter plots of NFs between V_0.15_ and V_min_-based scheme (*r* = Spearman rank correlation coefficient). **d** The comparison of target gene expression levels (CNRQ) using either the V_0.15_ or V_min_-based scheme. NF, normalization factor; CNRQ, calibrated normalized relative quantity

In the chondrogenic sub-study, the V_0.15_ was 0.079, while the V_min_ was 0.057 **(**Fig. 4a). Hence, combining the sequencing of eight candidate reference genes based on the M-value, the V_0.15_-based reference gene set contained *Gapdh* and *Rplp0*, and the V_min_-based reference gene set contained *Rplp0*, *Gapdh*, *Actb*, *Polr2e* and *Tbp* **(**Fig. 4b). The correlation coefficient in the Spearman rank correlation analysis between NF_V0.15_ and NF_Vmin_ was 0.833. This result showed that there was a rare correlation between these two selection schemes **(**Fig. 4c). The chondrogenic-related target genes, including *Acan*, *Sox9*, *Tgf-β*_*1*_ and *Tgf-β*_*3*_, were normalized to the two reference gene sets. The relative expression levels of *Acan*, *Sox9*, *Tgf-β*_*1*_ and *Tgf-β*_*3*_ showed a significant deference between the V_0.15_- and V_min_- based CNRQ values (*P* < 0.05), revealing that here the different selection schemes have a significant effect on the eventual normalization of target genes **(**Fig. 4d).

### V_0.20_/Opt3 vs. V_min_-based normalization scheme for analyzing gene expression data in adipose tissue

Three selection schemes were adopted in terms of the adipose tissue, either in the osteogenic or the chondrogenic sub-study including optimally three reference genes (Opt_3_), V_0.20_ and V_min_ schemes. The correlation of NF values between the V_0.20_ and V_min_ schemes with Opt_3_ and V_min_ schemes were analyzed including the variance of target genes expression levels generated by these three schemes was compared.

**Fig. 5:**
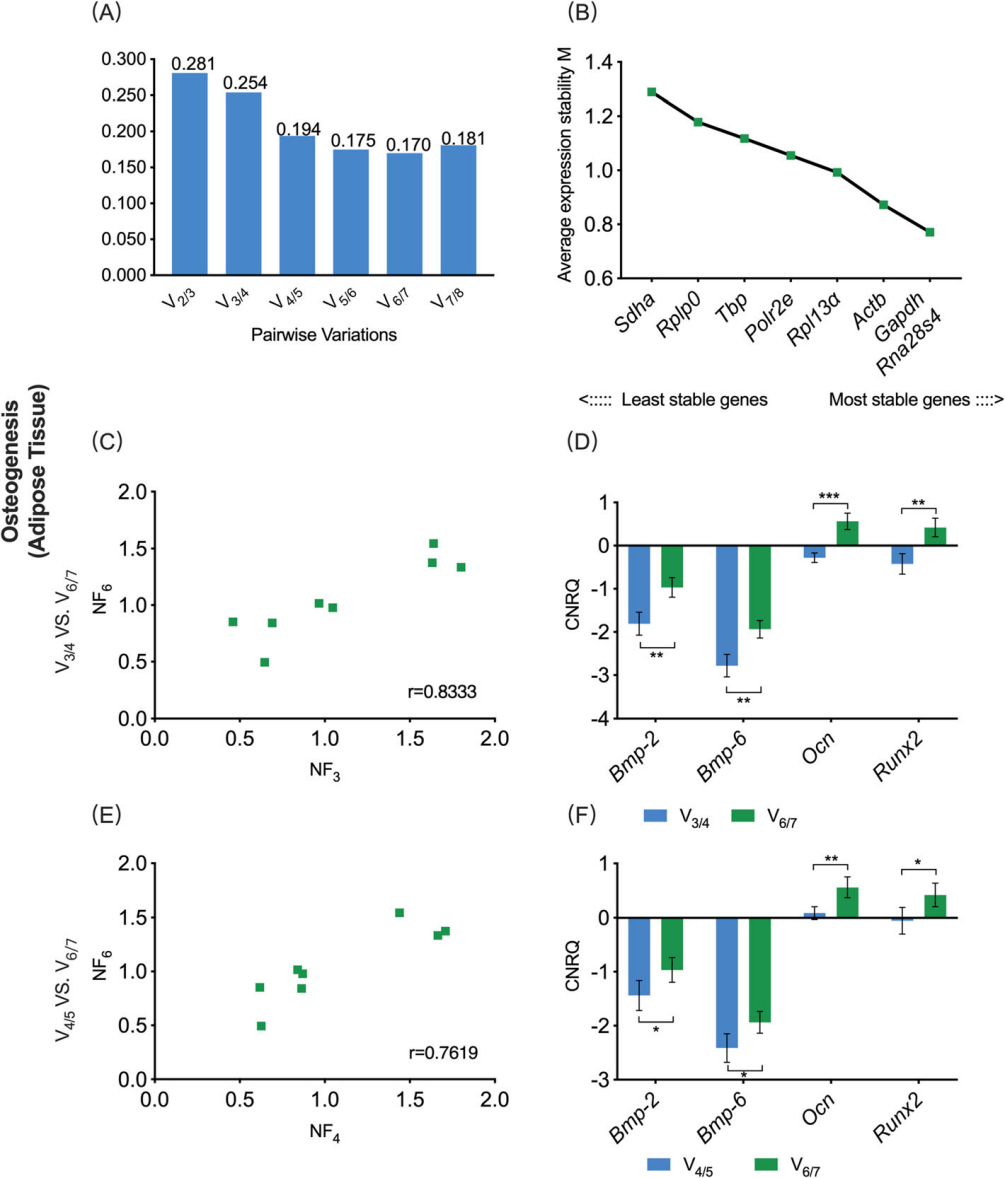
V_0.20_/Opt_3−_ vs. V_min_-based normalization schemes for gene expression assay on adipose tissue undergoing osteogenic differentiation. **a** Pairwise variation (V_n/n + 1_) analysis between NF_n_ and NF_n + 1_ to determine the optimal number of reference genes required for accurate normalization, V_0.20_ = 0.194, V_3/4_ = 0.254, V_min_ = 0.170; **b** The sequencing of eight candidate reference genes based on M value; **c**, **e** Scatter plots of NFs using three schemes (*r* = Spearman rank correlation coefficient). **d**, **f** The comparison of target gene expression levels (CNRQ) between V_0.20_/Opt3- and V_min_-based normalization schemes. NF, normalization factor; CNRQ, calibrated normalized relative quantity

In the osteogenic adipose sub-study, V_0.20_ was 0.194, while the V_min_ was 0.170 **(**Fig. 5a). Hence, combining the sequencing of eight candidate reference genes based on the M-value, the Opt_3_-based reference gene set contained *RNA 28S ribosomal 4 (Rna28s4)*, *Gapdh* and *Actb*, the V_0.20_-based reference gene set contained *Rna28s4*, *Gapdh*, *Actb* and *Rpl13α*, while the V_min_-based reference gene set contained *Rna28s4*, *Gapdh*, *Actb*, *Rpl13α*, *Polr2e* and *Tbp* **(**Fig. 5b). The *r*-value in the Spearman rank correlation analysis between NF_opt3_ and NF_Vmin_ was 0.833, with that of NF_V0.20_ and NF_Vmin_ was 0.762 **(**Fig. 5c, e**)**. These results showed that there was neither a correlation between V_min_ and V_0.20_ schemes nor between V_min_ and Opt3 schemes. The osteogenic-related target genes, including *Bmp-2*, *Bmp-6*, *Ocn* and *Runx2*, were normalized by three reference gene sets. The relative expression levels of *Bmp-2*, *Bmp-6*, *Ocn* and *Runx2* all showed significant deference between the Opt3- and V_min_- based CNRQ values including between the V_0.20_- and V_min_- based CNRQ values (*P* < 0.05), which revealed that different selection schemes have significant effects on the eventual normalization of target genes **(**Fig. 5d, f**)**.

**Fig. 6:**
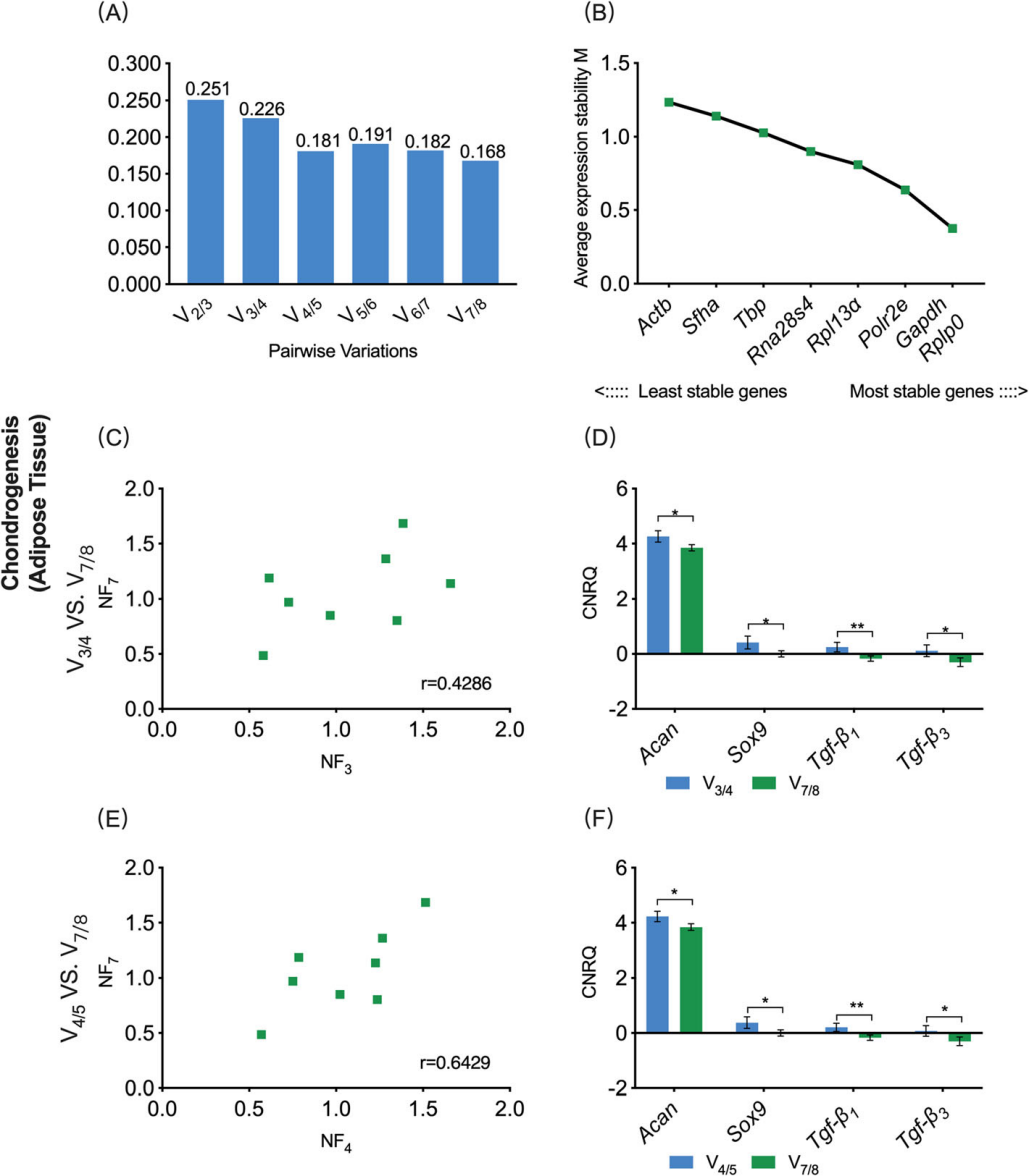
V_0.20_/Opt_3−_ vs. V_min_-based normalization schemes for gene expression assay on adipose tissue undergoing chondrogenic differentiation. **a** Pairwise variation (V_n/n + 1_) analysis between NF_n_ and NF_n + 1_ to determine the optimal number of reference genes required for accurate normalization, V_0.20_ = 0.181, V_3/4_ = 0.226, V_min_ = 0.168; **b** The sequencing of eight candidate reference genes based on M value; **c**, **e** Selected scatterplots of normalization factors using three schemes (*r* = Spearman rank correlation coefficient). **d**, **f** The comparison of target gene expression levels (CNRQ) between V_0.20_/_Opt3-_ and V_min-_based normalization schemes. NF, normalization factor; CNRQ, calibrated normalized relative quantity

In the chondrogenic adipose sub-study, V_0.20_ was 0.181, while the V_min_ was 0.168 **(**Fig. 6a**)**. Hence, combining the sequencing of eight candidate reference genes based on M value, the Opt_3_-based reference gene set contained *Rplp0*, *Gapdh* and *Polr2e*, the V_0.20_-based reference gene set contained *Rplp0*, *Gapdh*, *Polr2e* and *Rpl13α*, while the V_min_-based reference gene set contained *Rplp0*, *Gapdh*, *Polr2e*, *Rpl13α*, *Rna28s4*, *Tbp* and *succinate dehydrogenase complex flavoprotein subunit A (Sdha)* **(**Fig. 6b**)**. The *r*-value in the Spearman rank correlation analysis between NF_opt3_ and NF_Vmin_ was 0.4286, whilst between NF_V0.20_ and NF_Vmin_ was 0.6429 **(**Fig. 6c, e**)**. These results showed that there was neither a correlation between V_min_ and V_0.20_ schemes nor between V_min_ and Opt3 schemes. The chondrogenic-related target genes, including *Acan*, *Sox9*, *Tgf-β*_*1*_ and *Tgf-β*_*3*_, were normalized by three reference gene sets, and the CNRQ values were obtained. The relative expression levels of *Acan*, *Sox9*, *Tgf-β*_*1*_ and *Tgf-β*_*3*_ showed significant deference between the V_opt3_- and V_min_- based CNRQ values or between the V_0.20_- and V_min_- based CNRQ values (*P* < 0.05), which revealed that different selection schemes have significant effect on the eventual normalization of target genes **(**Fig. 6d, f**)**.

## Discussion

Accurate gene analysis remains essential for objective evaluation of the efficacy of bioengineering [31, 32]. Although the establishment of MIQE Guidelines and the advocacy of multiple reference genes has increased the reliability of the results [9, 15], there are still no unified solutions for the choice of the quantity and priority of reference genes, making much of the results across the bioengineering spectrum inconsistent [33, 34]. Several mathematical algorithms have been developed to solve this dilemma intended at detecting the stability of reference genes [14, 35, 36], such as geNorm [15], NormFinder [14], and BestKeeper [37]. In the present study, we as such sought to optimize the choice in geNorm schemes for reference genes determination where normally 0.15, commonly regarded as the cut-off point in the pairwise variation analysis, is a dangerous generalization as mono-cell cultures versus tissue-based experiments show clear deviations when using V_min_ or V_0.15_.

By integrating the results of geNorm, NormFinder, and BestKeeper, Tong et al. [38] and Zhang et al. [12] suggested that they had identified suitable reference gene(s) for normalization in gene expression with regards to their own specific experiments. It seems that the combination of different algorithms is a popular method [12, 14, 37, 38]. However, in our previous study, we indicated that the algorithms utilized were irrelevant with regard to the stability of reference genes evaluation; in other words, the different programs may not affect which reference genes should be selected and the optimum number needed to generate accurate results [23]. GeNorm remains one of the most accessible platforms in which a user-friendly interface, intuitive data presentation and subsequent infinite sample inclusion including candidate reference genes, make highly popular compared to the other algorithms [15]. Whilst one of the limitations of geNorm considers that co-related genes could occupy adjacent positions in the ranking, possibly causing a selection bias in the pairwise measurements [23, 38], our results keep validating the opposite in which the consistent stability of reference genes was maintained when these were removed successively. This indicates there is no co-regulation in the genes selected (data not shown). It was of critical importance to apply the mathematical algorithm accurately, which was the same purpose of this study and geNorm was considered a reliable and convenient method for analyzing the stability and determining the optimal number of the reference genes in a specific research endeavor [15, 23].

In terms of geNorm, the M-value reflects the stability of each reference gene, where the lowest value embodies the most stable expression, while the determination of the optimal number of reference genes for accurate gene expression analysis relies on the pattern of pairwise variation, the V-score [9]. The usual V-curve showed that adding reference genes is a double-edged sword in the normalization process. Whilst the nonspecific variation was eliminated by adding the stable reference genes and proven by the decrease in V-score, the increase after the minimum V-score indicated that unstable reference genes could interfere with the normalization process. Hence, it was reasonable that the participation of more reference genes after the minimum V-score do not contribute to minimizing the instability and variation of the reference gene set. In the initial design, Spearman rank correlation analysis was used to analyze the correlation of NF and then showed the pairwise variation (V_n/n + 1_) to assist determining the number of reference genes required for accurate normalization, in which the high correlation coefficients corresponded to the low V-scores [15]. Vandesompele et al. [15] decided to take 0.15 as the cut-off, below which the high correlation suggested that it was not necessary to include more reference genes. Indeed, our results of rBMSCs in the present study showed that the high correlation of the NFs obtained from two different gene sets, determined by the V_0.15_ and V_min_ (*r* = 0.95 for chondrogenesis; 0.97 for osteogenesis), respectively, confirmed the previous suggestion. However, the analysis of osteogenic induced muscle fragments presented a relatively low correlation coefficient when comparing the NF_V0.15_ with NF_Vmin_ (*r* = 0.83 for chondrogenesis; 0.64 for osteogenesis), which suggested that the variation caused by the additional reference genes only contained in the scheme based on V_min_ was significant. Erkens et al. [39] and Pérez et al. [30] all attempted to evaluate the V_min_-based scheme and they acknowledged that normalization became more accurate, but the improvement was minimal. Unfortunately, no corresponding verifications were performed in their reports, such as Spearman rank correlation analysis. Ragni et al. [40] also noticed the differences caused by diverse reference gene sets, but the analysis method used was to observe whether significant changes in the target genes occurred when different combinations of reference genes were applied for normalization, which supplied our experiments with a new evaluation method. The relative expression levels of chondrogenic- and part of osteogenic-related genes in our muscle tissue study showed significant differences when normalized by two reference gene sets (V_min_- or V_0.15_-based), but not in the milieu of all target gene when using rBMSCs. The results revealed that the variation erased by V_min_ based sets cannot be neglected in muscle tissue, which was consistent with the outcomes in Spearman rank correlation analysis. On the basis of the current data, regarding rBMSCs, the selection of reference genes based on V_0.15_ is fully viable. However, when using muscle tissue, the conditions need be stricter, where we recommend using the V_min_ scheme to obtain a more optimized set for normalization.

Interestingly, the pairwise variation analysis in the adipose tissue showed that none of the V-score was lower than 0.15, causing the V_0.15_ based scheme inapplicable. Facing a similar dilemma, Ayers et al. [25] flexibly elevated the threshold to 0.2, coincidentally, where the V-score corresponding to the decided number of reference genes was the minimum one in their analysis results. In a bovine adipose tissue explants study, Hosseini et al. [26] also drew on this new cut-off (V_0.2_) when determining the optimal reference genes due to the lack of values lower than 0.15. In the subsequent Spearman rank correlation analysis, although involving more reference genes provided a more stable normalization (*r* = 0.93), compared with using less reference genes based on the cut-off of 0.2 (*r* = 0.85), Hosseini et al. assumed it as “marginal” and hence ignored this difference [26]. In our study, the correlation between two NFs which corresponded to the V_min_ and V_0.2_ schemes were also weak, suggesting that V_min_, rather than a fixed threshold (e.g. 0.2), was still a reliable choice when all V-scores were higher than 0.15. Additionally, only choosing the three best reference genes was a compromise recommended by Vandesompele et al. [15] and Lu et al. [41], which we consider improper as according to the correlation analysis or the comparison of the relative expression levels of target genes in our adipose-related study the accuracy of normalization can be significantly improved when using V_min_ based reference genes, highlighting the advantages of the minimum scheme once again.

It is worth considering why different reference gene sets have significantly diverse performance in tissue fragments while keeping stable in rBMSCs, which may rely on the inherent difference between cells and tissue. Research conducted by Vandesompele et al. [15] also showed that the participation of more reference genes was required to remove non-specific variations in the normalization of genes in a tissue study. Compared with specific cell culture systems tissue models are more complex in which the heterogeneity of various cell types generates a multifaceted response when exposed to a single stimulus [42, 43]. Studies have shown that for different cell types, the expression of reference genes was not as stable [23, 44], not to mention when multiple reference genes and numerous cell types required to be weighed simultaneously, the change of a single factor, such as the number of reference genes, probably led to significant difference. Ren et al. [42] demonstrated that the response to osteogenic stimulation in muscle fragment with or without fascia was significantly different, suggesting that endogenous gene expression induced by the integration of external stimuli would change with the increase of cell types or tissue structures. Furthermore, concerning the RNA extraction, it was difficult for cells in tissues to be isolated independently from the extracellular matrix, while the influence of mRNA and protein in the matrix during the purification resulted in the enhancement of differences [45]. Homogenization was another potential threat, which is essential for RNA extraction from tissue models. However, the reduction of total RNA due to inadequate grinding, the limitation of the number of samples per homogenization and local temperature changes due to the high shearing forces could lead to loss, inactivation and degeneration of unstable cellular components [46].

## Conclusions

From the present study, we recommend that the proposed 0.15 cut-off value, according to the geNorm algorithm, should be carefully considered as cell and tissue experiments show clear variations. Whilst our results presently only reflect this for skeletal muscle and adipose tissue, some of the favorite tissues used in bioengineering experiments, whether this is applicable to all tissues needs to be further verified. However, from the results we can with certainty state that if the stability and variation of candidate reference genes, in a specific study are unclear, we recommend that the minimum V-score should be used as this provides a superior selection for determining the optimal number and category of reference genes needed to generate accurate and reproducible gene expression results. If not, we fear that the issue generating superior and critically reproducible gene expression results accurately will remain problematic, continuing to mislead the bioengineering field in developing reliable working applications that clinically are needed to bioengineer lost or damaged tissue types.

## Methods

### Study setup

**Fig. 7:**
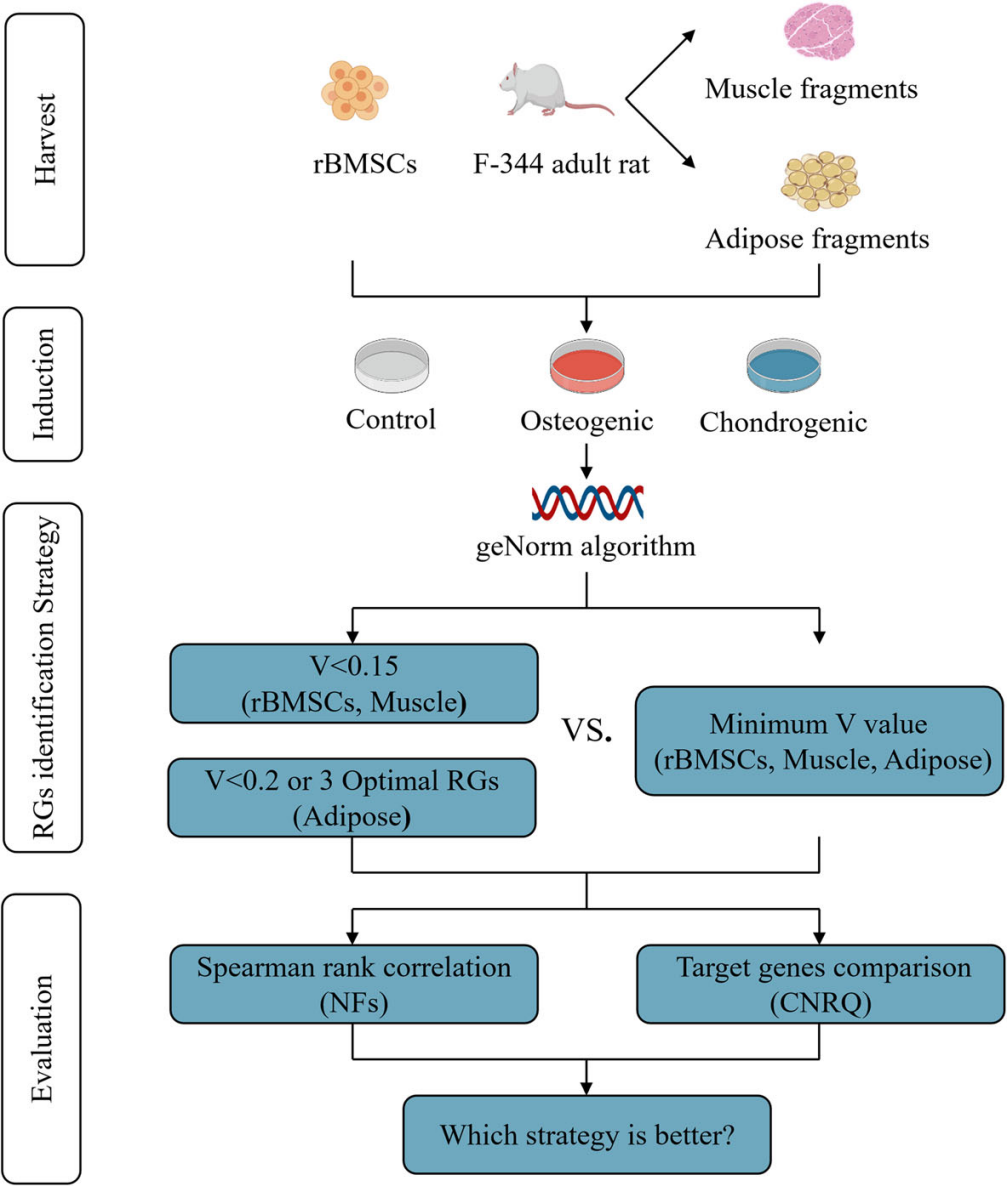
Schematic diagram on choosing the optimal normalization scheme for identifying proper reference genes. rBMSCs, rat bone marrow mesenchymal stem cells; RGs, reference genes; NF, normalization factor; CNRQ, calibrated normalized relative quantity

In the present study, either osteogenic or chondrogenic induction was applied to three commonly used cell or tissue types in bone and cartilage bioengineering: rBMSCs, rat skeletal muscle and adipose tissue. Eight candidate reference genes and osteogenic or chondrogenic related target genes were examined by RT-qPCR. Subsequently, the stability and pairwise variance of candidate reference genes were analyzed using geNorm [15]. The reference gene sets identified by different schemes would produce different NF values and different normalization results. The correlation of NF values and the variance of CNRQ values were performed to prove the variance between different schemes, then to define the optimal one for reference genes identification **(**Fig. 7**)**.

### Cell and tissue specimens

RBMSCs (passage 0, Sciencell, Carlsbad, CA, USA), skeletal muscle and adipose tissue from four F-344 adult female rats (Charles River Laboratories Wilmington, MA, USA) were used in this study. A total of 12 specimens per cell or tissue type were used with 4 specimens acting as the untreated control and the remaining 8 specimens being treated to either undergo chondrogenic (*n* = 4) or osteogenic differentiation (*n* = 4).

### Skeletal muscle tissue and adipose tissue harvest

For the tissue part of the study, a single F-344 adult female rat was sacrificed using an overdose of isoflurane (Abbot, Chicago, IL, USA). All practical experimental steps were performed in keeping with the rules and regulations of the Animal Protection Laboratory Animal Regulations (2013) and approved by the Animal Care Committee of Renji Hospital (Shanghai, China, No.: 201606230235). Under sterile conditions, fresh abdominal muscle and subcutaneous adipose tissue was harvested and placed temporarily in Dulbecco’s modified Eagle’s medium (DMEM; Biochrom Ltd., Cambridge, United Kingdom) containing high concentrations of Penicillin/Streptomycin (9%, P/S, Biochrom GmbH). Muscle (*n* = 8) and adipose (*n* = 8) tissue fragments were then collected using a 5 mm diameter biopsy punch (PFM medical, Cologne, Germany) and transferred into 24-well Nunc well culturing plates (Thermo Fisher Scientific, Waltham, MA, USA) in recovery medium consisting of DMEM supplemented with 3% P/S for 48 h at 37 °C containing 5% CO_2_ being treated to either undergo chondrogenic (*n* = 4) or osteogenic differentiation (*n* = 4). Fresh muscle and adipose tissue fragments (*n* = 4) were also collected as these would serve as the endogenous normalization control to which all tissue samples would be compared to.

### Cell culture

RBMSCs were used for the cellular culturing part, which were seeded at a density of 2*10^4^ per monolayer flask (Thermo Fisher Scientific) and cultured in DMEM supplemented with 3% P/S at 37 °C containing 5% CO_2_ for the primary culture. When cells reached 80% of confluence, they were detached using trypsin–EDTA (Biochrom Ltd), washed and submitted to new monolayer flasks at the same density for the sub-culture in the same manner thereafter. Cell morphology was observed under the light microscope, and photographs were taken. Cell numbers were counted at each cell passaged. Once 2nd passage cells reached 80% of confluence, they were passaged for the following induction of differentiation procedures. Some of the rBMSCs in 2nd passage pure without culturing were collected immediately as these would be used as the endogenous normalization control in downstream analysis procedures.

### Chondrogenic and osteogenic differentiation

To stimulate chondrogenic or osteogenic differentiation in both tissue and cell types the relevant media were utilized. The chondrogenic differentiation medium consisted of normal growth medium supplemented with 10 ng/mL recombinant BMP-6 (R&D Systems, Minneapolis, MN, USA), 10 ng/mL recombinant TGF-β_3_ (R&D Systems), 100 nM dexamethasone (Sigma-Aldrich, St. Louis, MI, USA), 50 μg/mL L-ascorbic acid-2-phosphate (Sigma-Aldrich), 40 μg/mL L-proline (Sigma-Aldrich), ITS+ 1(10 mg/L insulin, 5.5 mg/L transferrin, 4.7 μg/mL linoleic acid, 0.5 mg/mL bovine serum albumin, and 5 μg/L selenium) (Sigma-Aldrich) [32, 47–49]; the osteogenic differentiation medium consisted of normal growth medium supplemented with 50 μg/mL L-ascorbic acid-2-phosphate (Sigma-Aldrich), 1 mM L-glutamine (Sigma-Aldrich), 10 mM β-glycerophosphate (Sigma-Aldrich) and 100 nM dexamethasone (Sigma-Aldrich) [50]; the normal medium was DMEM supplemented with 3% P/S.

After 48 h recovery, harvested tissue specimens were collected, allocated randomly and then cultured in either chondrogenic differentiation (*n* = 4 per tissue type) or the osteogenic medium (*n* = 4 per tissue type), with normal recovery medium (*n* = 4 per tissue type) acting as the experimental control group. Tissue fragments were cultured for 7 days, medium changed every 2 days, collected and snap frozen in liquid nitrogen and then stored at − 80 °C until further use within 4 weeks.

Similarly, rBMSCs (passage 2) once having reached 80% confluence were trypsinized and seeded at 2*10^4^ cells per culture flask. Chondrogenesis (*n* = 4) or osteogenesis (*n* = 4) was then induced by utilizing the corresponding chondrogenic or osteogenic differentiation medium, respectively. Normal medium (*n* = 4) acted as the experimental control. The medium was changed every 2 days, and 7 days later the cells were harvested, immersed in trizol (Ambion, Carlsbad, CA, USA) and stored at − 80 °C for downstream analysis procedures.

### Primer design and optimization

Candidate reference genes were selected out of a gene library pool, known to be suitable for the optimization of reference genes in RT-qPCR, all with a standard deviation of the average amplification threshold cycle quantification value (Cq) less than 1 across 35 in rat tissues [51, 52]. Out of the candidate reference genes pool, the following eight genes were selected as candidates: *Rpl13α*, *Gapdh*, *Tbp*, *Rna28s4*, *Polr2e*, *Actb*, *Rplp0*, and *Sdha*. To study mRNA expression of the genes implicated in chondrogenesis, four chondrogenic-related genes were selected including *Acan*, *Sox9*, *Tgf-β*_*1*_ and *Tgf-β*_*3*_. Meanwhile, osteogenic-related genes included *Bmp-2, Bmp-6, Ocn and Runx2*. Primer sequences were designed utilizing PrimeQuest in conjunction with OligoAnalyzer 3.1 (https://eu.idtdna.com/site) and cross-referenced using the Basic Local Alignment Search Tool program (https://blast.ncbi.nlm.nih.gov/Blast.cgi) (see Additional file 1). All the primer sequences with relevant parameters were presented in Table 1.

**Table 1:** Gene primers for *Rattus norvegicus* with accession number, amplicon size and standard deviation in different cell/tissue types

	Gene	Accession Number	5′ sequence	3′ sequence	Amplicon length (bp)	Precision (Std.Dev.)
Reference genes	*Tbp*	BC081939.1	TAACCCAGAAAGTCGAAGAC	CCGTAAGGCATCATTGGA	185	0.02^a^/0.04^b^/0.13^c^
	*Gapdh*	BC083511.1	CATGGGTGTGAACCATGA	TGTCATGGATGACCTTGG	104	0.04/0.19/0.17
	*Polr2e*	BC158787.1	GACCATCAAGGTGTACTGC	CAGCTCCTGCTGTAGAAAC	151	0.08/0.16/0.13
	*Rplp0*	BC001834.2	CAACCCAGCTCTGGAGA	CAGCTGGCACCTTATTGG	116	0.10/0.05/0.36
	*Sdha*	NM_130428.1	GCGGTATGACACCAGTTATT	CCTGGCAAGGTAAACCAG	239	0.09/0.07/0.08
	*Rpl13α*	NM_173340.2	TTTCTCCGAAAGCGGATG	AGGGATCCCATCCAACA	159	0.16/0.03/0.11
	*Actb*	NM_031144.3	AGCTATGAGCTGCCTGA	GGCAGTAATCTCCTTCTGC	243	0.16/0.33/0.08
	*Rna28s4*	NR_145822.1	GCGGCCAAGCGTTCATA	CCTGTCTCACGACGGTCTAA	143	0.10/0.07/0.16
Genes of interest	*Tgf-β*_*1*_	NM_021578.2	TTTAGGAAGGACCTGGGTT	ACCCACGTAGTAGACGATG	210	0.04/0.11/0.11
	*Tgf-β*_*3*_	NM_013174.2	AACCTAAGGGTTACTATGCC	ACCACCATGTTGGACAG	214	0.06/0.06/0.08
	*Sox9*	NM_080403.1	CCAGAGAACGCACATCAAG	GGTGGTCGGTGTAGTCATA	161	0.07/0.12/0.08
	*Runx2*	NM_001278484.2	CCCAAGTGGCCACTTAC	CTGAGGCGGTCAGAGA	118	0.18/0.10/0.32
	*Acan*	NM_022190.1	CAAGTGGAGCCGTGTTT	GAGCGAAGGTTCTGGATTT	156	0.01/0.13/0.04
	*Bmp-6*	NM_013107.1	GGACATGGTCATGAGCTTTG	GTCAGAGTCTCTGTGCTGAT	232	0.43/0.08/0.22
	*Bmp-2*	NM_017178.1	GGAAGTGGCCCACTTAGA	TCACTAGCAGTGGTCTTACC	113	0.08/0.09/0.01
	*Ocn*	NM_013414.1	ACCTGGCAGGTGCAAA	CTCACACACCTCCCTGTG	237	0.03/0.08/0.11

^a^BMSCs, ^b^muscle tissue, ^c^adipose tissue. *Tbp* TATA-binding protein, *Gapdh* Glyceraldehyde 3-phosphate dehydrogenase, *Polr2e* RNA polymerase II subunit e, *Rplp0* Ribosomal protein lateral stalk subunit P0, *Sdha* Succinate dehydrogenase complex flavoprotein sub-unit A, *Rpl13α* Ribosomal protein L13α, *Actb* Actin beta, *Rna28s4* RNA 28S ribosomal 4, *Tgf-β*_*1*_ transforming growth factor, beta 1, *Tgf-ß3* transforming growth factor, beta 3, *Sox9* SRY (Sex Determining Region Y)-Box 9, *Runx2* Runt-related transcription factor 2, *Acan* Aggrecan, *Bmp-6* Bone morphogenetic protein 6, *Bmp-2* Bone morphogenetic protein 2, *Ocn* Osteocalcin

As previously established [23], primers were then stringently assessed for sequence amplification specificity with the annealing temperature predetermined to best function at 60 °C. A melt curve was included in each run to confirm amplification of a single product. After PCR amplification wells identified with positive amplicons underwent purified using the Mini Elute PCR Purification Kit (Qiagen, Crawley, UK) and analyzed, after Sanger sequencing (GATC Biotech, Cologne, Germany) utilizing BLASTN (https://blast.ncbi.nlm.nih.gov/Blast.cgi? PROGRAM = blastn&PAGE_TYPE = BlastSearch&.

LINK_LOC = blasthome), against the GenBank database (https://www.ncbi.nlm.nih.gov/genbank/) to validate primer reference gene sequence amplification specificity (see Additional file 2).

### RT-qPCR and geNorm assessment

Cells and muscle tissue samples were homogenized by liquid nitrogen in conjunction with a mortar and pestle. For all adipose tissue samples, a Micro-Dismembrator S (Sartorius Stedim Biotech, Göttingen, Germany) was utilized to homogenize. Subsequently, the RNeasy Fibrous Tissue Mini Kit (Qiagen, Hilden, Germany) was used to extract total RNA following the manufacturer’s protocol and eliminate DNAse and RNAse. Total extracted RNA concentration was determined spectrophotometrically at A_260/280_ with a NanoDrop™ Lite (Thermo Scientific, Waltham, USA) and RNA quality was assessed with a Pico6000 RNA kit (Agilent Technologies, Santa Clara, CA, USA) on a Bioanalyzer 2100 (Agilent Technologies). RNA integrity numbers were 8 for the cells and 7.8 for the muscle tissue and 7.5 for the adipose tissue. Reverse transcription was conducted using the QuantiTect Reverse Transcription cDNA Synthesis Kit (Qiagen, Hilden, Germany). Negative results of non-reverse transcription control run were confirmed and cDNA were stored at − 20 °C until use within 4 weeks.

The RT-qPCR was then performed in duplicate with FastStart Essential DNA Green Master (Roche, Basel, Switzerland) in a LightCycler® 96 thermocycler (Roche, Basel, Swiss). The total volume per reaction was 10 μL containing 2 μL cDNA (5 ng/μL), 5 μL FastStart Essential DNA Green Master (Roche), 0.6 μL forward primer and 0.6 μL reverse primer (10 μmol/L stock) and 1.8 μL RNase-free water. Cycling parameters including a pre-incubation of 3 min at 95 °C, followed by a three-step amplification program of 40 cycles consisting of a denaturation, annealing and extension step set at 95 °C for 10 s, 60 °C for 15 s and 72 °C for 30 s, respectively; each gene in either BMSCs, muscle tissue or adipose tissue also included a standard curve for quality purposes (see Supplementary Table 1, Additional file 3). No cq value for the no template control was detected.

The relative quantity of all the candidate reference genes were detected in all samples including the rBMSCs, adipose and muscle tissue with or without chondrogenic or osteogenic induction. The geNorm algorithm (http://medgen.ugent.be/wjvdesomp/geNorm/) was used to evaluate the stability and priority of these candidate reference genes [53]. The raw Cq values of each genes in each sub-study were pre-processed by 2^ΔCq^ algorithm, then the generated data was inputted into geNorm. After the matrix was loaded, a table containing NF of each reference gene was produced, followed by two charts. The first chart showed the sequence of gene stability, in which the stability was improved from left to right, as shown by the decrease of M value. A gene with M < 1.5 is considered as a stable reference gene [15]. The second chart determined the recommended number of the reference genes being used for a specific study, which was indicated by the V_n/n + 1_-score. Here, two schemes were compared. Firstly, according to the geNorm algorithm [15], the value of V_n/n + 1_ under 0.15 indicating that no additional reference genes are required for normalization was set as the control scheme. In certain cases, where no V_n/n + 1_-score was less than 0.15, the Opt_3_ or V_0.20_ were considered as alternatives. Secondly, V_min_ was set as the cut-off for choosing the optimal quantity of reference genes.

### The relative quantity of osteogenic- or chondrogenic-related target genes

The normalization of each target gene was accomplished by qbase plus software version 3.0 (Biogazelle, Zwijnaarde, Belgium-www.qbaseplus.com), and the results were presented as CNRQ value, which reflect the relative quantity of each target gene based on the selected reference gene set. Upon different schemes, different reference gene sets were used and subsequently different relative quantities of a certain target gene were obtained. All CNRQ values were scaled to the endogenous control that were pure untreated muscle and adipose tissue including rBMSCs.

### Statistics

Normalization factors obtained by different schemes from geNorm were analyzed in GraphPad Prism (GraphPad software Version 5, San Diego, CA) using Spearman rank correlation (correlations with *P* < 0.05 were considered significant; correlations were very strong when Spearman’s rank correlation coefficient (r) was greater than 0.9). A two-tailed unpaired t-test in GraphPad Prism was used to determine whether different selection schemes of reference genes had significant effects on the normalization of relative expression levels of a certain gene. *P* < 0.05 values were considered significantly different.

## Supplementary Information

**Additional file 1.** RT-qPCR target in silico specificity and location data.

**Additional file 2.** RT-qPCR product sequencing data.

**Additional file 3.** Standard Curves of BMSCs, muscle tissue, and adipose tissue.

**Additional file 4: Supplementary Table 1.** RT-qPCR validation data.

## Data Availability

The necessary algorithmic codes of the program GeNorm are readily available at (https://www.qbaseplus.com/modal_forms/nojs/login). All data, raw and processed, is readily available from the corresponding author on request.

## Abbreviations

RT-qPCR: Reverse transcription quantitative real-time polymerase chain reaction
MIQE: the Minimum Information for Publication of Quantitative Real-Time PCR Experiments
NF: Normalization factor
rBMSCs: rat bone marrow mesenchymal stem cells
*Rpl13α*: *Ribosomal protein L13α*
*Actb*: *Actin beta*
*Polr2e*: *rna polymerase II subunit e*
*Bmp*: *Bone morphogenetic protein*
*Ocn*: *Osteocalcin*
*Runx2*: *Runt-related transcription factor 2*
CNRQ: Calibrated normalized relative quantity
*Acan*: *Aggrecan*
*Sox9*: *Sex determining region Y-box 9*
*Tgf-β*: *Transforming growth factor beta*
*Gapdh*: *Glyceraldehyde 3-phosphate dehydrogenase*
*Tbp*: *TATA-binding protein*
*Rplp0*: *Ribosomal protein lateral stalk subunit P0*
Opt3: Optimal three reference genes
*Rna28s4*: *RNA 28S ribosomal 4*
*Sdha*: *Succinate dehydrogenase complex flavoprotein subunit A*
DMEM: Dulbecco’s modified Eagles’ medium
P/S: Penicillin/streptomycin
Cq: Cycle quantification value
