# Supplementary material for: Optimizations for identifying reference genes in bone and cartilage bioengineering
Source: BMC Biotechnol. 2021 Mar 17;21:25. doi: 10.1186/s12896-021-00685-8 (PMC7972220; doi:10.1186/s12896-021-00685-8)
Supplement: Supplementary file 1 — Additional file 1. RT-qPCR target in silico specificity and location data. [file 12896_2021_685_MOESM1_ESM.pdf]

Additional file 1. RT-qPCR Target *In silico* specificity and location data.

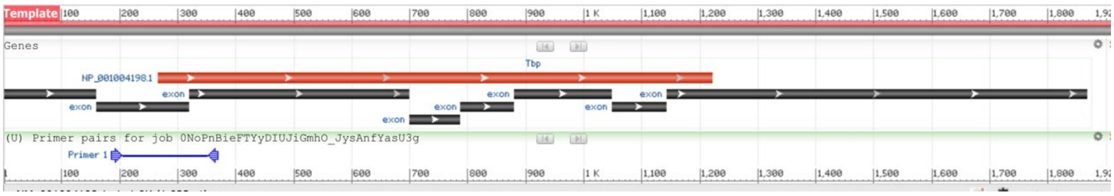

Tbp

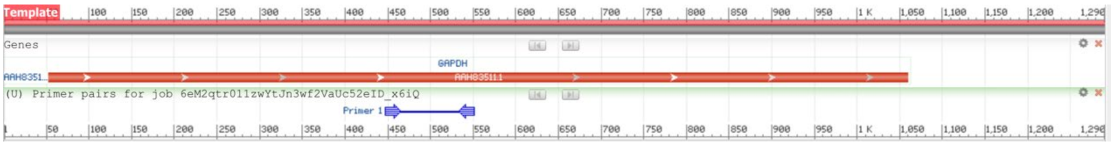

Gapdh

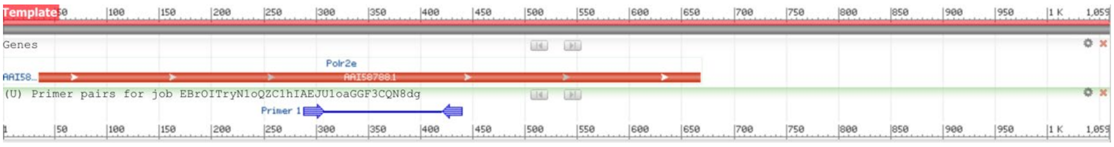

Polr2e

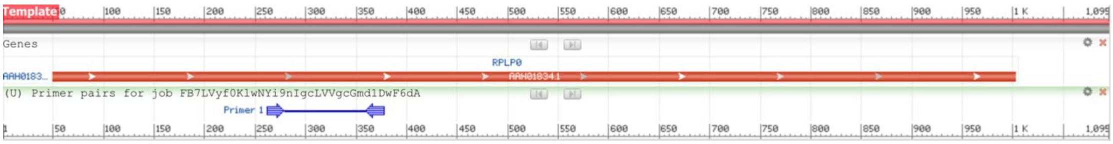

Rplp0

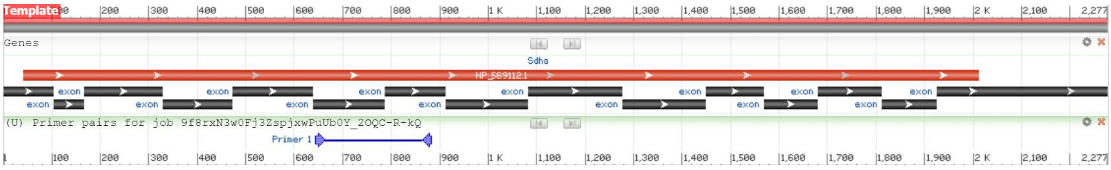

Sdha

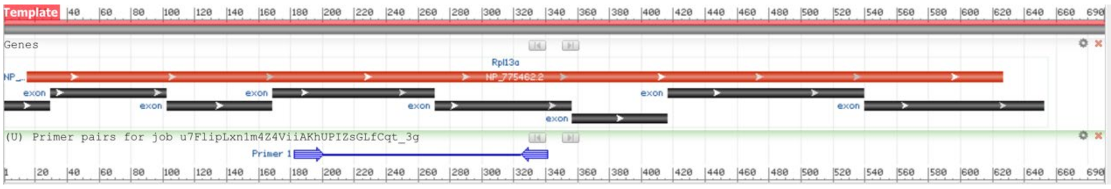

Rpl13α

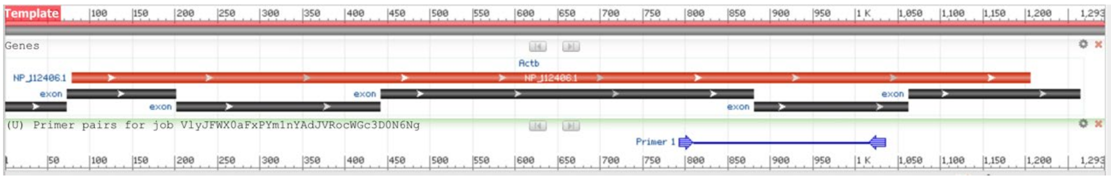

Actb

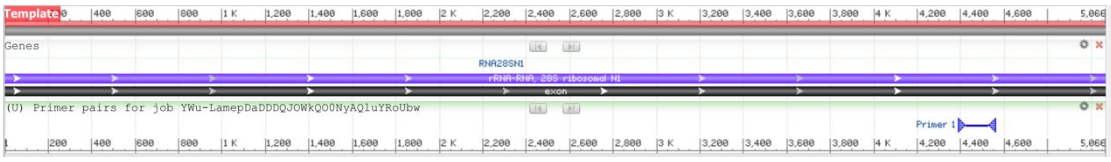

Rna28s4

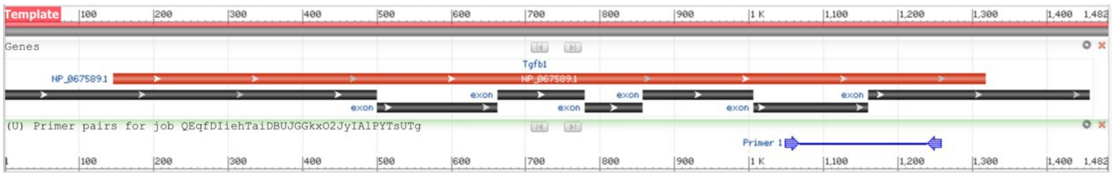

Tgf-β_1

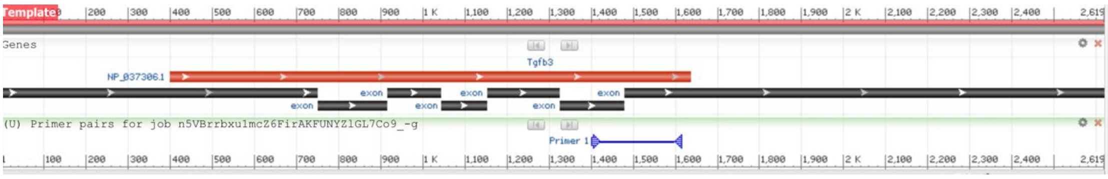

Tgf-β_3

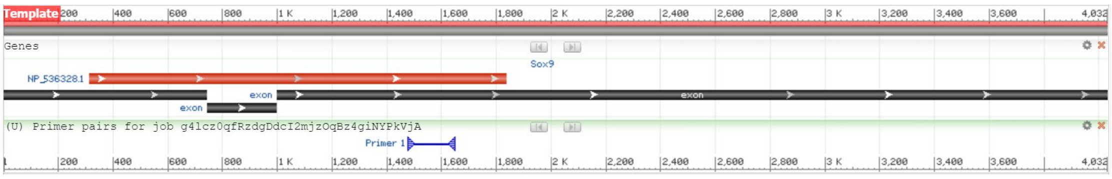

Sox9

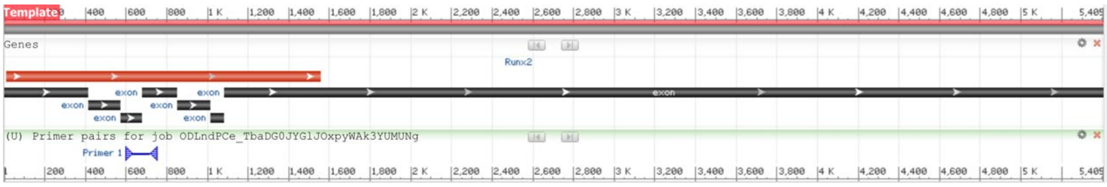

Runx2

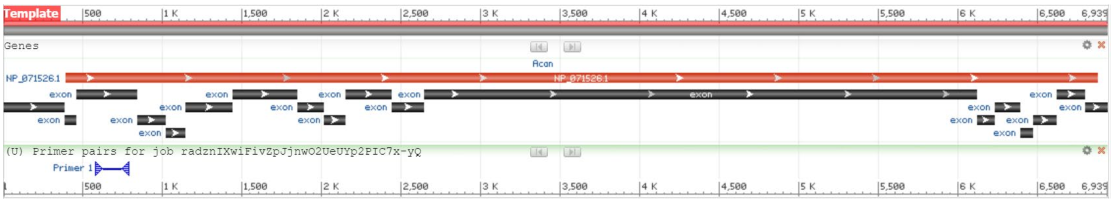

Acan

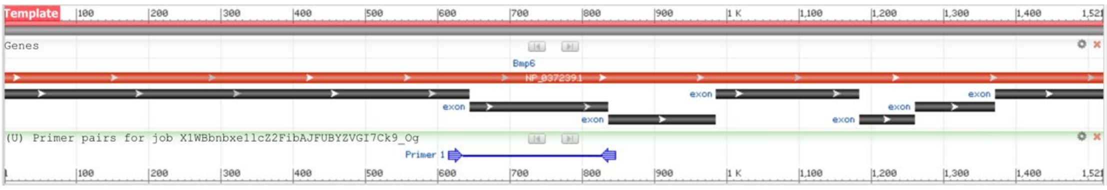

Bmp-6

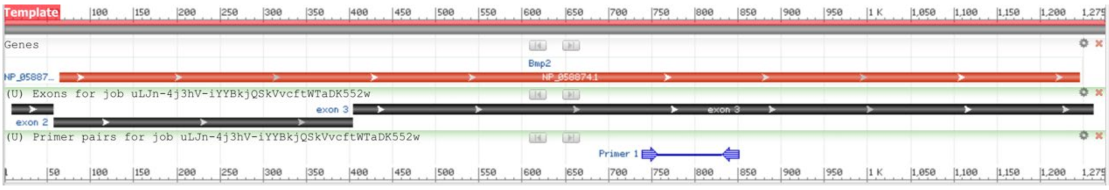

Bmp-2

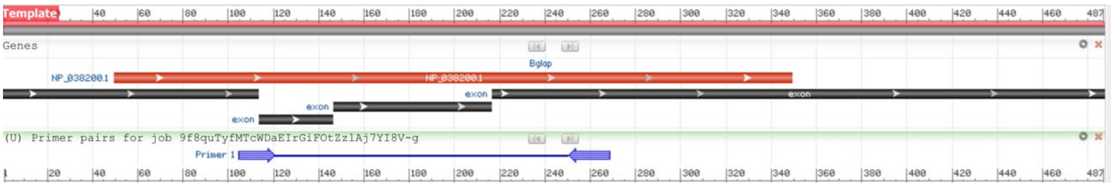
Template
Genes
Bglap
NP_038200.1
NP_038200.1
exon
exon
exon
exon
(U) Primer pairs for job 9f8quTyfMTcWDaEIrGiFOtZzlAj7YI8V-g
Primer 1

Ocn
