# Supplementary material for: Optimizations for identifying reference genes in bone and cartilage bioengineering
Source: BMC Biotechnol. 2021 Mar 17;21:25. doi: 10.1186/s12896-021-00685-8 (PMC7972220; doi:10.1186/s12896-021-00685-8)
Supplement: Supplementary file 2 — Additional file 2. RT-qPCR product sequencing data. [file 12896_2021_685_MOESM2_ESM.pdf]

Additional file 2. RT-qPCR validation data.

Graphic Summary

Distribution of the top 10 Blast Hits on 10 subject sequences

Mouse over to see the title, click to show alignments

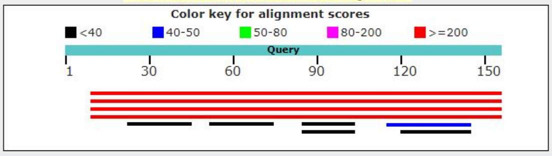

Descriptions

Sequences producing significant alignments:

Select: All None Selected:0

Alignments Download GenBank Graphics Distance tree of results

Description	Max score	Total score	Query cover	E value	Ident	Accession
PREDICTED: Rattus norvegicus TATA box binding protein (Tbp), transcript variant X3, mRNA	250	250	94%	4e-65	98%	gi|1046840159|XM_006227980.3
PREDICTED: Rattus norvegicus TATA box binding protein (Tbp), transcript variant X1, mRNA	250	250	94%	4e-65	98%	gi|1046840158|XM_008758747.2
PREDICTED: Rattus norvegicus TATA box binding protein (Tbp), transcript variant X2, mRNA	250	250	94%	4e-65	98%	gi|672034386|XM_008758748.1

Tbp

Graphic Summary

Distribution of the top 10 Blast Hits on 10 subject sequences

Mouse over to see the title, click to show alignments

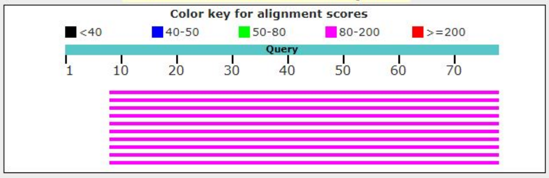

Descriptions

Sequences producing significant alignments:

Select: All None Selected:0

Alignments Download GenBank Graphics Distance tree of results

Description	Max score	Total score	Query cover	E value	Ident	Accession
PREDICTED: Rattus norvegicus glyceraldehyde-3-phosphate dehydrogenase (Gapdh), transcript variant X1, mRNA	114	114	89%	2e-22	97%	gi|1046883605|XM_017592435.1
PREDICTED: Rattus norvegicus glyceraldehyde-3-phosphate dehydrogenase (LOC108351137), mRNA	114	114	89%	2e-22	97%	gi|1046832236|XM_017602936.1
PREDICTED: Rattus norvegicus similar to glyceraldehyde-3-phosphate dehydrogenase (RGD1562758), misc_RNA	114	114	89%	2e-22	97%	gi|1046826450|XR_598347.2
Rattus norvegicus glyceraldehyde-3-phosphate dehydrogenase (Gapdh), mRNA	114	114	89%	2e-22	97%	gi|402691727|NM_017008.4

Gapdh

Graphic Summary

Distribution of the top 10 Blast Hits on 10 subject sequences

Mouse over to see the title, click to show alignments

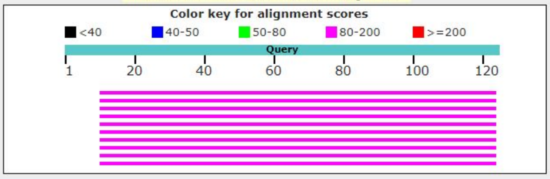

Descriptions

Sequences producing significant alignments:

Select: All None Selected:0

Alignments Download GenBank Graphics Distance tree of results

Description	Max score	Total score	Query cover	E value	Ident	Accession
PREDICTED: Rattus norvegicus polymerase (RNA) II subunit E (Polr2e), transcript variant X1, mRNA	199	199	91%	1e-47	99%	gi|1046897107|XM_006241009.3
PREDICTED: Rattus norvegicus polymerase (RNA) II subunit E (Polr2e), transcript variant X3, mRNA	199	199	91%	1e-47	99%	gi|564358321|XM_006241011.1
PREDICTED: Rattus norvegicus polymerase (RNA) II subunit E (Polr2e), transcript variant X2, mRNA	199	199	91%	1e-47	99%	gi|564358319|XM_006241010.1

Polr2e

Graphic Summary

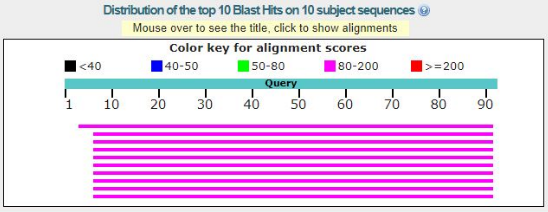

Descriptions

Sequences producing significant alignments:

Select: All None Selected:0

Alignments Download GenBank Graphics Distance tree of results

	Description	Max score	Total score	Query cover	E value	Ident	Accession
☐	Rattus norvegicus TL0ADA46YP06 mRNA sequence	145	145	95%	1e-31	98%	gi|298881191|FQ229722.1
☐	Rattus norvegicus ribosomal protein lateral stalk subunit P0 (Rplp0), mRNA	143	143	92%	4e-31	98%	gi|310616731|NM_022402.2
☐	Rattus norvegicus TL0ABA29YC07 mRNA sequence	143	143	92%	4e-31	98%	gi|298916696|FQ210412.1
☐	Rattus norvegicus TL0ACA39YL23 mRNA sequence	143	143	92%	4e-31	98%	gi|298916498|FQ216913.1

Rplp0

Graphic Summary

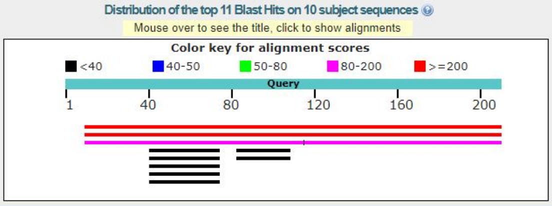

Descriptions

Sequences producing significant alignments:

Select: All None Selected:0

Alignments Download GenBank Graphics Distance tree of results

	Description	Max score	Total score	Query cover	E value	Ident	Accession
☐	Rattus norvegicus succinate dehydrogenase complex subunit A (SDHA) mRNA, partial cds	352	352	95%	6e-96	99%	gi|89574166|DQ402976.1
☐	Rattus norvegicus succinate dehydrogenase complex flavoprotein subunit A (Sdha), mRNA	352	352	95%	6e-96	99%	gi|18426857|NM_130428.1
☐	Rattus norvegicus CH230-2P9 (Children's Hospital Oakland Research Institute Rat (BN/SsNHsd/MCW) BAC library) complete sequence	181	357	95%	2e-44	98%	gi|226514538|AC094217.7

Sdha

Graphic Summary

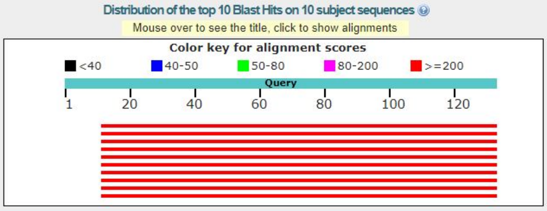

Descriptions

Sequences producing significant alignments:

Select: All None Selected:0

Alignments Download GenBank Graphics Distance tree of results

	Description	Max score	Total score	Query cover	E value	Ident	Accession
☐	PREDICTED: Rattus norvegicus ribosomal protein L13A (Rpl13a), transcript variant X1, mRNA	219	219	91%	1e-53	100%	gi|1046842905|XM_017589309.1
☐	Rattus norvegicus TL0ADA19YK18 mRNA sequence	219	219	91%	1e-53	100%	gi|298915883|FQ222814.1
☐	Rattus norvegicus TL0ADA47YG07 mRNA sequence	219	219	91%	1e-53	100%	gi|298914584|FQ229596.1
☐	Rattus norvegicus TL0ADA47YK12 mRNA sequence	219	219	91%	1e-53	100%	gi|298914501|FQ229513.1
☐	Rattus norvegicus TL0ADA47YL04 mRNA sequence	219	219	91%	1e-53	100%	gi|298914487|FQ229499.1

Rpl13α

Graphic Summary

Distribution of the top 10 Blast Hits on 10 subject sequences

Mouse over to see the title, click to show alignments

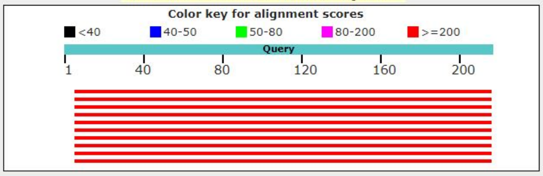

Descriptions

Sequences producing significant alignments:

Select: All None Selected:0

Alignments Download GenBank Graphics Distance tree of results

	Description	Max score	Total score	Query cover	E value	Ident	Accession
☐	Rattus norvegicus actin, beta (Actb), mRNA	363	363	97%	8e-97	99%	gi|402744873|NM_031144.3
☐	Rattus norvegicus TL0AEA64YM19 mRNA sequence	363	363	97%	8e-97	99%	gi|298914700|FQ232682.1
☐	Rattus norvegicus TL0ADA48YH01 mRNA sequence	363	363	97%	8e-97	99%	gi|298911322|FQ229293.1
☐	Rattus norvegicus TL0AEA64YO15 mRNA sequence	363	363	97%	8e-97	99%	gi|298911110|FQ232650.1
☐	Rattus norvegicus TL0AEA67YC11 mRNA sequence	363	363	97%	8e-97	99%	gi|298907175|FQ232063.1

Actb

Graphic Summary

Distribution of the top 10 Blast Hits on 10 subject sequences

Mouse over to see the title, click to show alignments

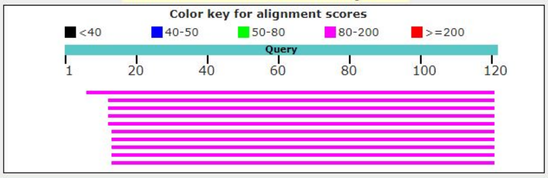

Descriptions

Sequences producing significant alignments:

Select: All None Selected:0

Alignments Download GenBank Graphics Distance tree of results

	Description	Max score	Total score	Query cover	E value	Ident	Accession
☐	Schistocephalus solidus genome assembly S_solidus_NST_G2, scaffold SSLN_scaffold0003238	196	196	94%	1e-46	98%	gi|689428055|LL903470.1
☐	Plagiostomum stellatum 28S ribosomal RNA gene, partial sequence	196	196	89%	1e-46	100%	gi|588313914|KC869872.1
☐	Plagiostomum whitmani 28S ribosomal RNA gene, partial sequence	196	196	89%	1e-46	100%	gi|588313913|KC869871.1
☐	Amastigomonas bermudensis 18S ribosomal RNA gene, partial sequence; internal transcribed spacer 1, 5.8S ribosomal RNA gene, and internal transcribe	196	196	89%	1e-46	100%	gi|300078505|GU001167.1

Rna28s4

Distribution of the top 10 Blast Hits on 10 subject sequences

Mouse over to see the title, click to show alignments

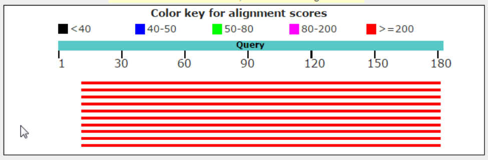

Descriptions

Sequences producing significant alignments:

Select: All None Selected:0

Alignments Download GenBank Graphics Distance tree of results

	Description	Max score	Total score	Query cover	E value	Ident	Accession
☐	Rattus norvegicus transforming growth factor, beta 1 (Tgfb1), mRNA	298	298	93%	2e-77	99%	gi|148747597|NM_021578.2
☐	Rattus norvegicus TGF beta 1 mRNA, complete cds	298	298	93%	2e-77	99%	gi|45239049|AY550025.1
☐	Rat mRNA for transforming growth factor-beta 1	298	298	93%	2e-77	99%	gi|57341|X52498.1
☐	PREDICTED: Mus caroli transforming growth factor beta 1 (Tgfb1), mRNA	277	277	93%	8e-71	96%	gi|1195729563|XM_021167684.1
☐	PREDICTED: Mus pahari transforming growth factor beta 1 (Tgfb1), transcript variant X2, mRNA	277	277	93%	8e-71	96%	gi|1195523161|XM_021219156.1
☐	PREDICTED: Mus pahari transforming growth factor beta 1 (Tgfb1), transcript variant X1, mRNA	277	277	93%	8e-71	96%	gi|1195523159|XM_021219155.1
☐	Mus musculus transforming growth factor, beta 1 (Tgfb1), mRNA	277	277	93%	8e-71	96%	gi|930697458|NM_011577.2
☐	Mus musculus transforming growth factor, beta 1, mRNA (cDNA clone MGC:5747 IMAGE:3586216), complete cds	277	277	93%	8e-71	96%	gi|15489274|BC013738.1
☐	Mus musculus B cells CRL-1702 WEHI 231 cDNA, RIKEN full-length enriched library, clone:G430055L01 product:transforming growth factor, beta 1, full insert sequence	277	277	93%	8e-71	96%	gi|74184430|AK144163.1
☐	Mus musculus mRNA for transforming growth factor-beta 1	277	277	93%	8e-71	96%	gi|3688423|AJ009862.1

Tgf-β_1

Distribution of the top 10 Blast Hits on 10 subject sequences

Mouse over to see the title, click to show alignments

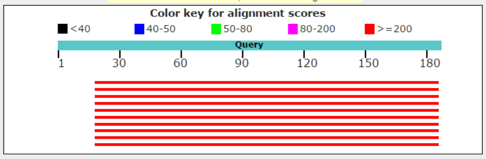

Descriptions

Sequences producing significant alignments:

Select: All None Selected:0

Alignments Download GenBank Graphics Distance tree of results

Description	Max score	Total score	Query cover	E value	Ident	Accession
PREDICTED: Rattus norvegicus transforming growth factor, beta 3 (Tgfb3), transcript variant X1, mRNA	302	302	89%	2e-78	100%	gi|564356731|XM_006240366.1
Rattus norvegicus transforming growth factor, beta 3 (Tgfb3), mRNA	302	302	89%	2e-78	100%	gi|292781870|NM_013174.2
Rattus norvegicus transforming growth factor, beta 3, mRNA (cDNA clone MGC:105479 IMAGE:7313608), complete cds	302	302	89%	2e-78	100%	gi|62089483|BC092195.1
Rattus norvegicus Wistar transforming growth factor beta-3 mRNA, complete cds	302	302	89%	2e-78	100%	gi|424131|U03491.1
Synthetic construct transforming growth factor beta 3 (Tgfb3) gene, complete cds	297	297	89%	8e-77	99%	gi|444189315|JX974603.1
Mus musculus transforming growth factor, beta 3 (Tgfb3), mRNA	257	257	89%	7e-65	94%	gi|225637539|NM_009368.3
Mus musculus transforming growth factor, beta 3, mRNA (cDNA clone IMAGE:30615448)	257	257	89%	7e-65	94%	gi|63101504|BC094591.1
Mus musculus 10 days neonate skin cDNA, RIKEN full-length enriched library, clone:4732489J19 product:transforming growth factor, beta 3, full insert sequence	257	257	89%	7e-65	94%	gi|74208940|AK132519.1
Mus musculus mammary gland RCB-0527 Jyg-MC(B) cDNA, RIKEN full-length enriched library, clone:G930044C01 product:transforming growth factor, beta 3, full insert sequence	257	257	89%	7e-65	94%	gi|74208309|AK145304.1
Mus musculus B16 F10Y cells cDNA, RIKEN full-length enriched library, clone:G370121J04 product:transforming growth factor, beta 3, full insert sequence	257	257	89%	7e-65	94%	gi|74203844|AK148385.1

Tgf-β3

Distribution of the top 100 Blast Hits on 100 subject sequences

Mouse over to see the title, click to show alignments

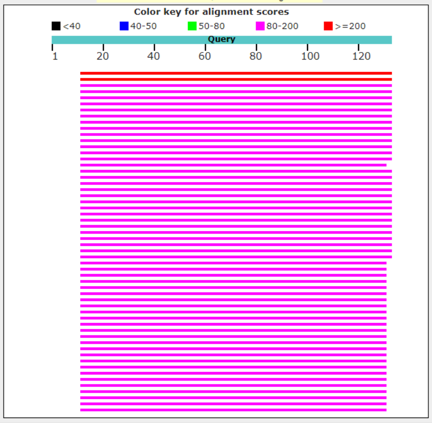

Descriptions

Sequences producing significant alignments:

Select: All None Selected:0

Alignments Download GenBank Graphics Distance tree of results

Description	Max score	Total score	Query cover	E value	Ident	Accession
Rattus norvegicus SRY (sex determining region Y)-box 9 (SRY) mRNA, complete cds	219	219	91%	1e-53	100%	gi|755223120|KP732536.1
Rattus norvegicus SRY box 9 (Sox9), mRNA	219	219	91%	1e-53	100%	gi|747[illegible]

Questions/comments

Sox9

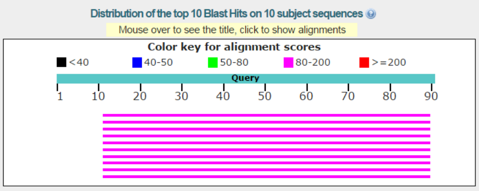

Descriptions

Sequences producing significant alignments:

Select: All None Selected:0

Alignments Download GenBank Graphics Distance tree of results

Description	Max score	Total score	Query cover	E value	Ident	Accession
PREDICTED: Mus pahari runt related transcription factor 2 (Runx2), transcript variant X2, mRNA	129	129	86%	1e-26	96%	gi|1195520331|XM_021217616.1
PREDICTED: Mus pahari runt related transcription factor 2 (Runx2), transcript variant X1, mRNA	129	129	86%	1e-26	96%	gi|1195520329|XM_021217615.1
PREDICTED: Rattus norvegicus runt-related transcription factor 2 (Runx2), transcript variant X7, mRNA	129	129	86%	1e-26	96%	gi|1046904680|XM_017596551.1
PREDICTED: Rattus norvegicus runt-related transcription factor 2 (Runx2), transcript variant X4, mRNA	129	129	86%	1e-26	96%	gi|1046904674|XM_006244551.3
PREDICTED: Rattus norvegicus runt-related transcription factor 2 (Runx2), transcript variant X3, mRNA	129	129	86%	1e-26	96%	gi|1046904673|XM_006244550.3
PREDICTED: Rattus norvegicus runt-related transcription factor 2 (Runx2), transcript variant X2, mRNA	129	129	86%	1e-26	96%	gi|1046904670|XM_017596547.1
PREDICTED: Rattus norvegicus runt-related transcription factor 2 (Runx2), transcript variant X1, mRNA	129	129	86%	1e-26	96%	gi|1046904668|XM_006244549.3
PREDICTED: Peromyscus maniculatus bairdii runt-related transcription factor 2-like (LOC102927917), mRNA	129	129	86%	1e-26	98%	gi|1008803488|XM_006997764.2
Rattus norvegicus Runx2 mRNA, runt related transcription factor 2, alternative splicing isoform 1 skipping exon2, partial sequence	129	129	86%	1e-26	96%	gi|803375177|AB573881.1
Rattus norvegicus runt-related transcription factor 2 (Runx2), transcript variant 2, mRNA	129	129	86%	1e-26	96%	gi|686661106|NM_001278484.2

Runx2

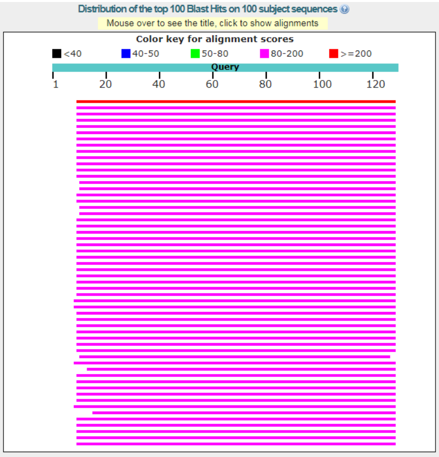

Descriptions

Sequences producing significant alignments:

Select: All None Selected:0

Alignments Download GenBank Graphics Distance tree of results

Description	Max score	Total score	Query cover	E value	Ident	Accession
Rattus norvegicus aggrecan (Acan), mRNA	215	215	92%	2e-52	100%	gi|11990615|NM_022190.1
PREDICTED: Mus caroli aggrecan (Acan), transcript variant X2, mRNA	192	192	92%	2e-45	96%	gi|1195[illegible]

Acan

Distribution of the top 23 Blast Hits on 20 subject sequences

Mouse over to see the title, click to show alignments

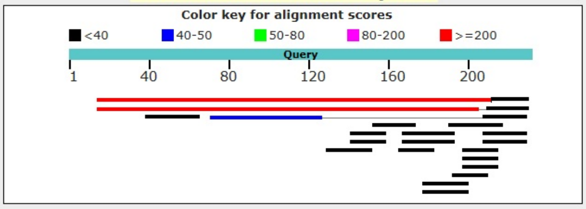

Descriptions

Sequences producing significant alignments:

Select: All None Selected:0

Alignments Download GenBank Graphics Distance tree of results

	Description	Max score	Total score	Query cover	E value	Ident	Accession
☐	Rattus norvegicus bone morphogenetic protein 6 (Bmp6), mRNA	298	337	93%	1e-79	95%	gi|29789037|NM_013107.1
☐	Rattus norvegicus bone morphogenetic protein-6 (BMP-6) mRNA, partial cds	288	327	91%	2e-76	95%	gi|1854674|U66298.1
☐	Rattus norvegicus bone morphogenetic protein 5 (Bmp5), mRNA	42.8	78.3	33%	0.015	77%	gi|157823366|NM_001108168.1

Bmp-6

Graphic Summary

Distribution of the top 10 Blast Hits on 10 subject sequences

Mouse over to see the title, click to show alignments

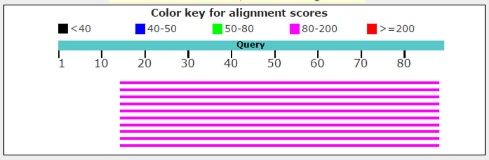

Descriptions

Sequences producing significant alignments:

Select: All None Selected:0

Alignments Download GenBank Graphics Distance tree of results

	Description	Max score	Total score	Query cover	E value	Ident	Accession
☐	PREDICTED: Rattus norvegicus bone morphogenetic protein 2 (Bmp2), transcript variant X1, mRNA	127	127	82%	3e-26	99%	gi|1046878540|XM_008762246.2
☐	Rattus norvegicus bone morphogenetic protein 2 (Bmp2), mRNA	127	127	82%	3e-26	99%	gi|8392989|NM_017178.1
☐	Rat bone morphogenetic protein 2 related mRNA sequence	127	127	82%	3e-26	99%	gi|310175|L20678.1
☐	PREDICTED: Mus pahari bone morphogenetic protein 2 (Bmp2), mRNA	114	114	82%	2e-22	95%	gi|1195537593|XM_021193195.1
☐	PREDICTED: Mesocricetus auratus bone morphogenetic protein 2 (Bmp2), transcript variant X2, mRNA	109	109	82%	9e-21	93%	gi|1196035704|XM_021226375.1
☐	PREDICTED: Mesocricetus auratus bone morphogenetic protein 2 (Bmp2), transcript variant X1, mRNA	109	109	82%	9e-21	93%	gi|1196035703|XM_005068676.3
☐	PREDICTED: Cricetulus griseus bone morphogenetic protein 2 (Bmp2), transcript variant X2, mRNA	109	109	82%	9e-21	93%	gi|1032963906|XM_007638750.2
☐	PREDICTED: Cricetulus griseus bone morphogenetic protein 2 (Bmp2), transcript variant X1, mRNA	109	109	82%	9e-21	93%	gi|1032829534|XM_003495268.3
☐	PREDICTED: Mus musculus bone morphogenetic protein 2 (Bmp2), transcript variant X1, mRNA	109	109	82%	9e-21	93%	gi|568915158|XM_006498619.1

Bmp-2

Graphic Summary

Distribution of the top 10 Blast Hits on 10 subject sequences

Mouse over to see the title, click to show alignments

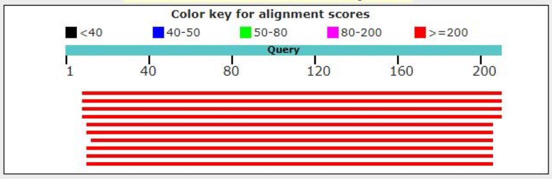

Descriptions

Sequences producing significant alignments:

Select: All None Selected:0

Alignments Download GenBank Graphics Distance tree of results

	Description	Max score	Total score	Query cover	E value	Ident	Accession
☐	PREDICTED: Rattus norvegicus bone gamma-carboxyglutamate protein (Bglap), transcript variant X1, mRNA	351	351	96%	5e-93	99%	gi|1046871701|XM_006232594.3
☐	Rattus norvegicus bone gamma-carboxyglutamate protein (Bglap), mRNA	351	351	96%	5e-93	99%	gi|11761542|NM_013414.1
☐	Rat mRNA for bone gla protein (BGP)	351	351	96%	5e-93	99%	gi|55826|X04141.1
☐	Rat BGP mRNA encoding gamma-carboxyglutamic acid (Gla) protein, complete cds	351	351	96%	5e-93	99%	gi|203147|M11777.1

Ocn
