# Supplementary material for: Optimizations for identifying reference genes in bone and cartilage bioengineering
Source: BMC Biotechnol. 2021 Mar 17;21:25. doi: 10.1186/s12896-021-00685-8 (PMC7972220; doi:10.1186/s12896-021-00685-8)

Standard Curve of MSCs

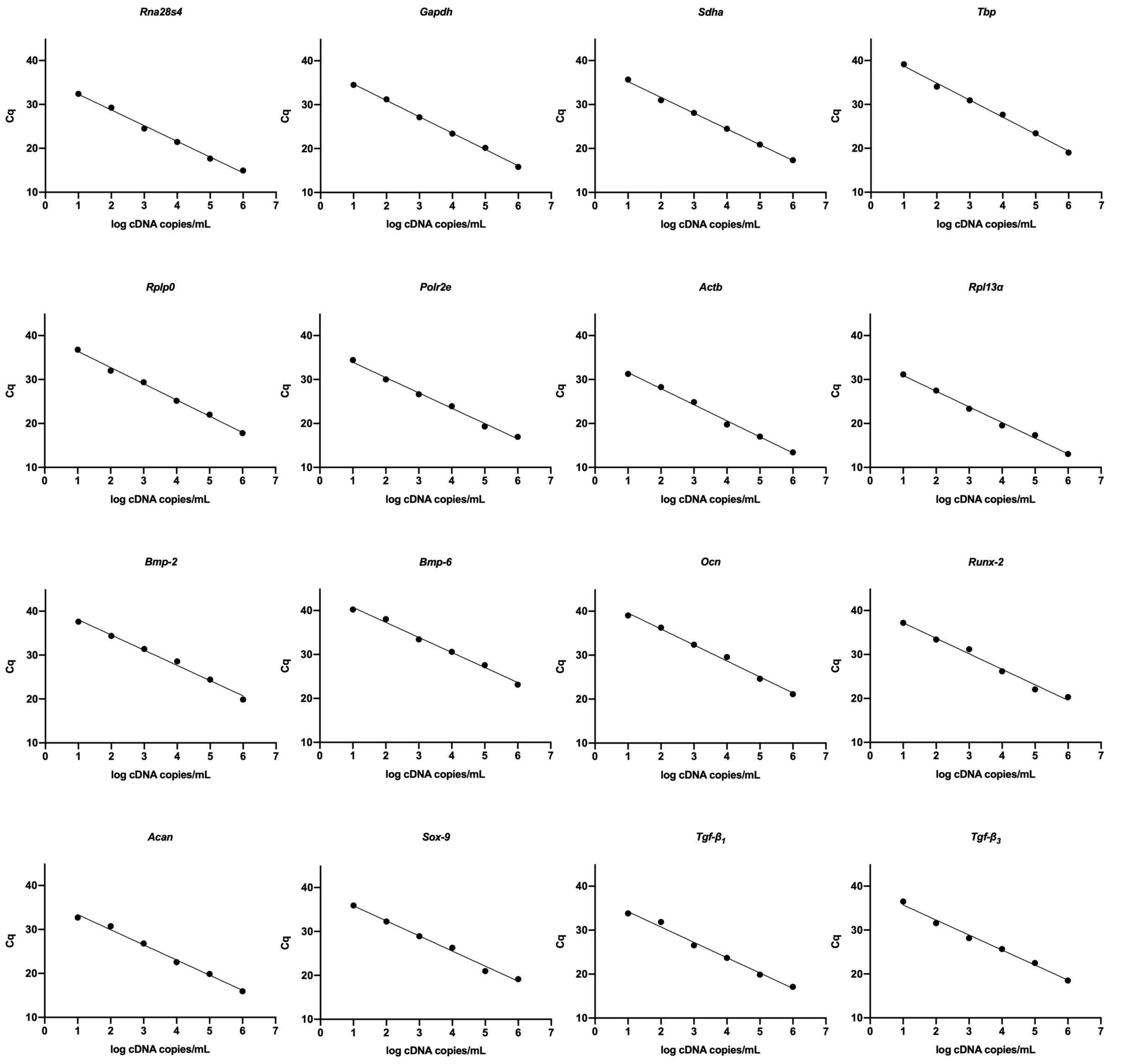

Standard Curve of Muscle Tissue

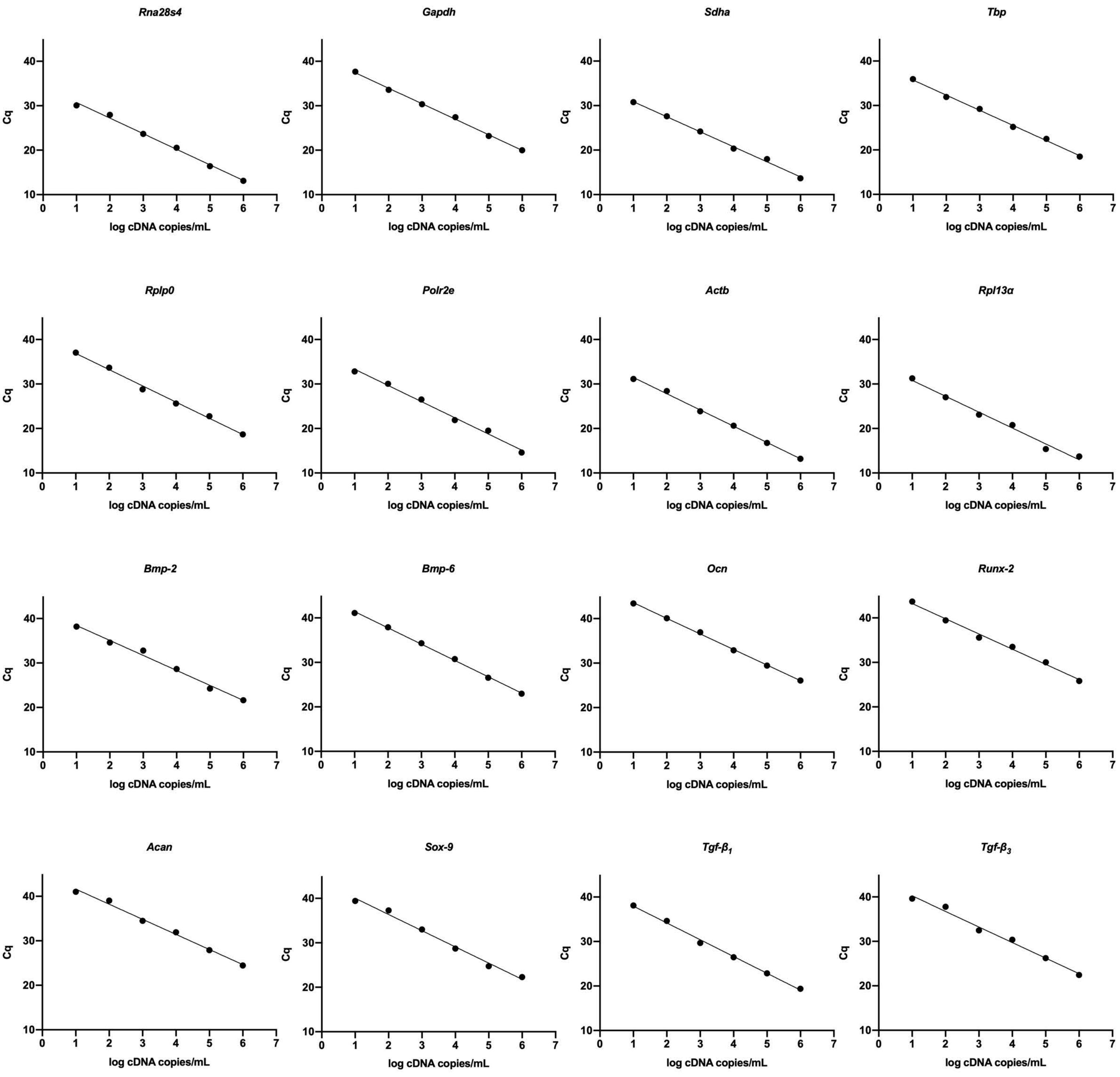

Standard Curve of Adipose Tissue

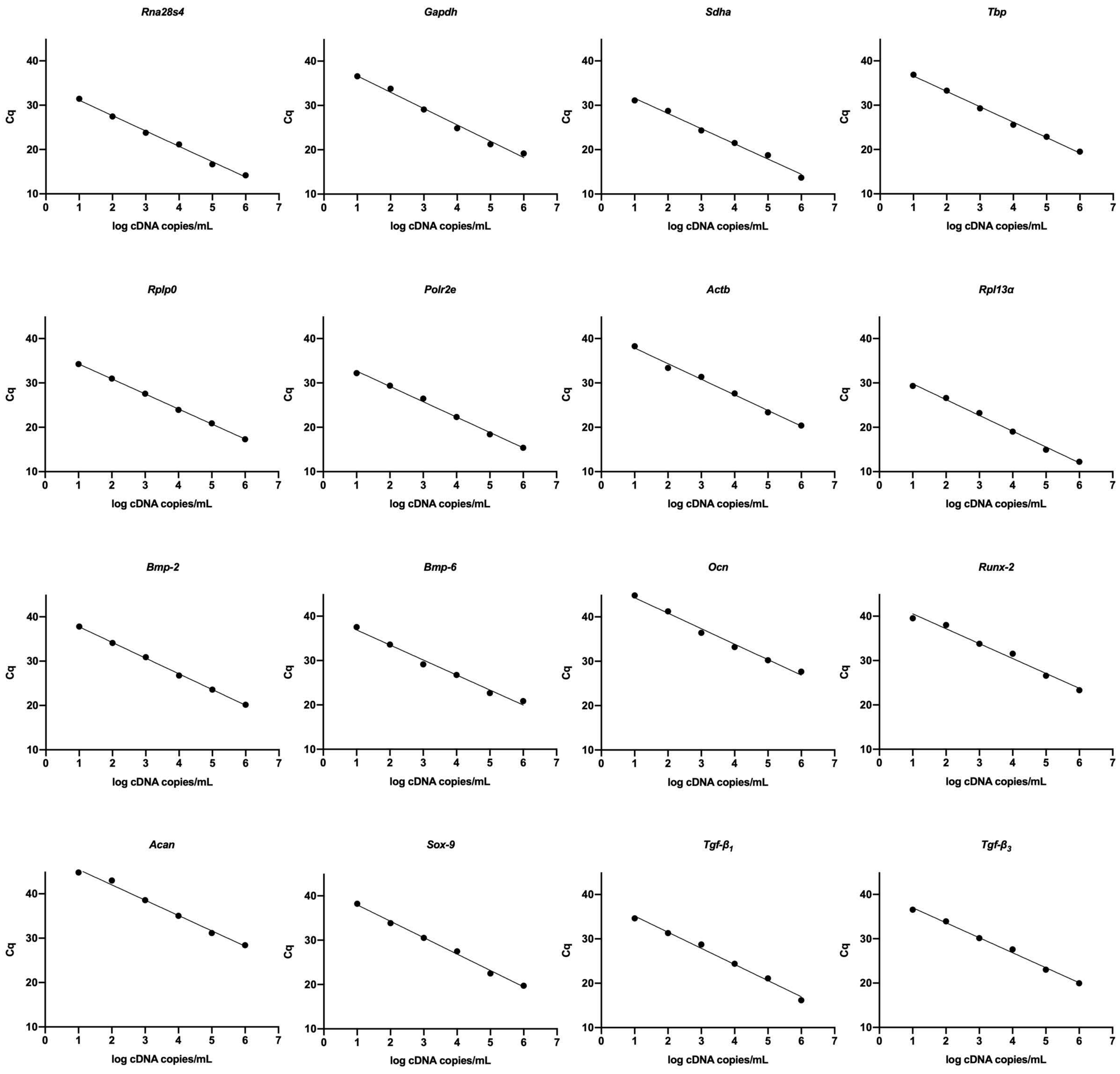

Supplement: Supplementary file 3 — Additional file 3. Standard Curves of BMSCs, muscle tissue, and adipose tissue. [file 12896_2021_685_MOESM3_ESM.pdf]
