# Supplementary material for: Optimizations for identifying reference genes in bone and cartilage bioengineering
Source: BMC Biotechnol. 2021 Mar 17;21:25. doi: 10.1186/s12896-021-00685-8 (PMC7972220; doi:10.1186/s12896-021-00685-8)
Supplement: Supplementary file 4 — Additional file 4: Supplementary Table 1. RT-qPCR validation data. [file 12896_2021_685_MOESM4_ESM.docx]

| Supplementary Table 1. RT-qPCR validation data. | | | | | | | | | | |  |
| --- | --- | --- | --- | --- | --- | --- | --- | --- | --- | --- | --- |
|  |  | E | %E | LOD | LOQ | Slope | Y-intercept | R^2^ | Linear dynamic range (log cDNA copies/mL) | Specificity |  |
| Bone marrow stromal cells | Rna28s4 | 1.90 | 90.37 | -0.46 | -1.39 | -3.58 | 35.89 | 0.9950 | 1 to 6 | Y |  |
|  | Gapdh | 1.86 | 85.57 | -0.24 | -0.71 | -3.72 | 38.41 | 0.9987 | 1 to 6 | Y |  |
|  | Sdha | 1.90 | 89.93 | -0.34 | -1.03 | -3.59 | 38.81 | 0.9972 | 1 to 6 | Y |  |
|  | Tbp | 1.81 | 81.18 | -0.45 | -1.38 | -3.88 | 42.61 | 0.9950 | 1 to 6 | Y |  |
|  | Rplp0 | 1.86 | 86.47 | -0.40 | -1.23 | -3.70 | 40.12 | 0.9960 | 1 to 6 | Y |  |
|  | Polr2e | 1.94 | 93.51 | -0.52 | -1.57 | -3.49 | 37.42 | 0.9935 | 1 to 6 | Y |  |
|  | Actb | 1.88 | 87.59 | -0.48 | -1.45 | -3.66 | 35.24 | 0.9945 | 1 to 6 | Y |  |
|  | Rpl13α | 1.91 | 90.72 | -0.47 | -1.43 | -3.57 | 34.47 | 0.9946 | 1 to 6 | Y |  |
|  | Bmp-2 | 1.94 | 94.31 | -0.61 | -1.86 | -3.47 | 41.50 | 0.9910 | 1 to 6 | Y |  |
|  | Bmp-6 | 1.96 | 95.97 | -0.55 | -1.68 | -3.42 | 44.15 | 0.9926 | 1 to 6 | Y |  |
|  | Ocn | 1.88 | 88.28 | -0.51 | -1.56 | -3.64 | 43.24 | 0.9937 | 1 to 6 | Y |  |
|  | Runx-2 | 1.92 | 92.04 | -0.75 | -2.28 | -3.53 | 40.74 | 0.9865 | 1 to 6 | Y |  |
|  | Acan | 1.95 | 94.94 | -0.56 | -1.71 | -3.45 | 36.85 | 0.9923 | 1 to 6 | Y |  |
|  | Sox-9 | 1.95 | 95.43 | -0.65 | -1.97 | -3.44 | 39.28 | 0.9899 | 1 to 6 | Y |  |
|  | Tgf-β_1_ | 1.93 | 93.15 | -0.65 | -1.97 | -3.50 | 37.72 | 0.9899 | 1 to 6 | Y |  |
|  | Tgf-β_3_ | 1.96 | 96.16 | -0.62 | -1.88 | -3.42 | 39.12 | 0.9908 | 1 to 6 | Y |  |
|  |  |  |  |  |  |  |  |  |  |  |  |
| Muscle tissue | Rna28s4 | 1.93 | 93.06 | -0.47 | -1.42 | -3.50 | 34.21 | 0.9947 | 1 to 6 | Y |  |
|  | Gapdh | 1.93 | 93.24 | -0.31 | -0.92 | -3.50 | 40.94 | 0.9977 | 1 to 6 | Y |  |
|  | Sdha | 1.98 | 97.61 | -0.37 | -1.13 | -3.38 | 34.26 | 0.9967 | 1 to 6 | Y |  |
|  | Tbp | 1.96 | 96.47 | -0.36 | -1.08 | -3.41 | 39.15 | 0.9970 | 1 to 6 | Y |  |
|  | Rplp0 | 1.88 | 87.93 | -0.45 | -1.38 | -3.65 | 40.53 | 0.9950 | 1 to 6 | Y |  |
|  | Polr2e | 1.88 | 88.32 | -0.55 | -1.66 | -3.64 | 36.97 | 0.9927 | 1 to 6 | Y |  |
|  | Actb | 1.88 | 87.76 | -0.33 | -0.99 | -3.66 | 35.13 | 0.9974 | 1 to 6 | Y |  |
|  | Rpl13α | 1.91 | 90.50 | -0.74 | -2.24 | -3.57 | 34.38 | 0.9870 | 1 to 6 | Y |  |
|  | Bmp-2 | 1.98 | 97.97 | -0.66 | -2.00 | -3.37 | 41.82 | 0.9896 | 1 to 6 | Y |  |
|  | Bmp-6 | 1.87 | 87.37 | -0.25 | -0.75 | -3.67 | 45.10 | 0.9985 | 1 to 6 | Y |  |
|  | Ocn | 1.93 | 92.84 | -0.20 | -0.61 | -3.51 | 47.08 | 0.9990 | 1 to 6 | Y |  |
|  | Runx-2 | 1.96 | 96.16 | -0.57 | -1.72 | -3.42 | 46.64 | 0.9922 | 1 to 6 | Y |  |
|  | Acan | 1.97 | 97.06 | -0.55 | -1.65 | -3.40 | 45.01 | 0.9928 | 1 to 6 | Y |  |
|  | Sox-9 | 1.88 | 88.02 | -0.61 | -1.86 | -3.65 | 43.67 | 0.9909 | 1 to 6 | Y |  |
|  | Tgf-β_1_ | 1.84 | 84.08 | -0.38 | -1.16 | -3.77 | 41.72 | 0.9964 | 1 to 6 | Y |  |
|  | Tgf-β_3_ | 1.93 | 92.84 | -0.69 | -2.09 | -3.51 | 43.75 | 0.9886 | 1 to 6 | Y |  |
|  |  | | | | | | | | | |  |
| Adipose tissue | Rna28s4 | 1.94 | 94.31 | -0.45 | -1.36 | -3.47 | 34.58 | 0.9952 | 1 to 6 | Y |  |
|  | Gapdh | 1.87 | 86.90 | -0.67 | -2.04 | -3.68 | 40.34 | 0.9891 | 1 to 6 | Y |  |
|  | Sdha | 1.96 | 95.84 | -0.66 | -1.99 | -3.43 | 35.01 | 0.9896 | 1 to 6 | Y |  |
|  | Tbp | 1.94 | 94.00 | -0.37 | -1.12 | -3.48 | 40.07 | 0.9967 | 1 to 6 | Y |  |
|  | Rplp0 | 1.97 | 97.24 | -0.13 | -0.39 | -3.39 | 37.69 | 0.9996 | 1 to 6 | Y |  |
|  | Polr2e | 1.95 | 94.54 | -0.43 | -1.29 | -3.46 | 36.14 | 0.9956 | 1 to 6 | Y |  |
|  | Actb | 1.92 | 92.49 | -0.56 | -1.71 | -3.52 | 41.38 | 0.9923 | 1 to 6 | Y |  |
|  | Rpl13α | 1.91 | 90.94 | -0.45 | -1.36 | -3.56 | 33.35 | 0.9951 | 1 to 6 | Y |  |
|  | Bmp-2 | 1.92 | 91.60 | -0.21 | -0.62 | -3.54 | 41.28 | 0.9990 | 1 to 6 | Y |  |
|  | Bmp-6 | 1.97 | 97.42 | -0.74 | -2.25 | -3.39 | 40.29 | 0.9869 | 1 to 6 | Y |  |
|  | Ocn | 1.93 | 93.42 | -0.67 | -2.04 | -3.49 | 47.81 | 0.9891 | 1 to 6 | Y |  |
|  | Runx-2 | 1.98 | 98.38 | -0.84 | -2.53 | -3.36 | 43.90 | 0.9834 | 1 to 6 | Y |  |
|  | Acan | 1.95 | 94.72 | -0.58 | -1.77 | -3.46 | 48.93 | 0.9918 | 1 to 6 | Y |  |
|  | Sox-9 | 1.86 | 86.25 | -0.44 | -1.34 | -3.70 | 41.67 | 0.9953 | 1 to 6 | Y |  |
|  | Tgf-β_1_ | 1.88 | 88.41 | -0.59 | -1.78 | -3.64 | 38.78 | 0.9917 | 1 to 6 | Y |  |
|  | Tgf-β_3_ | 1.98 | 97.51 | -0.47 | -1.42 | -3.38 | 40.38 | 0.9947 | 1 to 6 | Y |  |
| E : PCR efficiency = 10^(-1/slope); %E :%PCR efficiency= (E-1)*100; LOD: limit of detection = [3.3*(S.D.(intercept) / slope)]; LOQ: limit of quantitation = [10*(S.D.(intercept) / slope)]; Specificity: Evaluate the melt profiles when using SYBR®-based assays. For probe-based assays, gel analysis is required to observe a single band (one PCR product); *Tbp: TATA-binding protein; Gapdh: Glyceraldehyde 3-phosphate dehydrogenase; Polr2e: RNA polymerase II subunit e; Rplp0: Ribosomal protein lateral stalk subunit P0; Sdha: Succinate dehydrogenase complex flavoprotein sub-unit A; Rpl13α: Ribosomal protein L13 α; Actb: Actin beta; Rna28s4: RNA 28S ribosomal 4; Tgf-β1: transforming growth factor beta 1; Tgf-ß3, transforming growth factor beta 3; Sox9: Sex determining region Y-box 9; Runx2: Runt-related transcription factor 2; Acan: Aggrecan; Bmp-6: Bone morphogenetic protein 6; Bmp-2: Bone morphogenetic protein 2; Ocn: Osteocalcin.* | | | | | | | | | | |  |
|  |  |  |  |  |  |  |  |  |  |  |  |
|  |  |  |  |  |  |  |  |  |  |  |  |
|  |  |  |  |  |  |  |  |  |  |  |  |
|  |  |  |  |  |  |  |  |  |  |  |  |
|  |  |  |  |  |  |  |  |  |  |  |  |
